# Epigenetically Downregulated Breast Cancer Gene 2 through Acetyltransferase Lysine Acetyltransferase 2B Increases the Sensitivity of Colorectal Cancer to Olaparib

**DOI:** 10.3390/cancers15235580

**Published:** 2023-11-25

**Authors:** Siche Chen, Heike Allgayer

**Affiliations:** Department of Experimental Surgery—Cancer Metastasis, Mannheim Medical Faculty, Ruprecht Karls University of Heidelberg, 68167 Mannheim, Germany; siche.chen@stud.uni-heidelberg.de

**Keywords:** olaparib sensitivity, BRCA2, KAT2B, colorectal cancer, H3K27ac, γH2AX

## Abstract

**Simple Summary:**

We show that breast cancer gene 2 (*BRCA2*) is highly expressed in cultured olaparib-resistant colorectal cancer (CRC) cells and that CRC cells become more sensitive to olaparib with reduced BRCA2 expression. Importantly, inhibiting lysine acetyltransferase 2B (KAT2B) expression also decreases the resistance of CRC cells towards olaparib treatment. Mechanistically, a lack of KAT2B reduces the acetylation of histone H3 lysine 27 (H3K27), thus binding the latter to *BRCA2* promotors, which affects BRCA2 expression. Our research suggests the critical role of a KAT2B-BRCA2 axis, which is also valid for *BRCA2* wildtype phenotypes, opening interesting new perspectives for better-predicting olaparib sensitivity in CRC.

**Abstract:**

Olaparib suppresses DNA damage repair by inhibiting the poly ADP ribose polymerase (PARP), especially in cancers with *BRCA1/2* mutations or the BRCA-ness phenotype. However, the first trials showed that some patients with defective DNA damage repair are still resistant to olaparib. The recovery of the wildtype BRCA is a prominent mechanism of PARP inhibitor (PARPi) resistance in BRCA-deficient tumors, but additional molecular features of olaparib resistance remain poorly understood. The objective of our study was to find molecular parameters that contribute to olaparib response or resistance in CRC. We report that downregulating histone acetyltransferase KAT2B decreases BRCA2 expression by reducing the acetylation of the 27th amino acid in histone H3 (H3K27) at the promoter of the *BRCA2* gene in colorectal cancer (CRC). This increases the sensitivity of CRC cells toward olaparib treatment. The H3K27ac binding domain of *BRCA2* may be required for its transcription. Low endogenous KAT2B expression, which we identify in a subset of cultured BRCA2-expressing CRC cells, leads to an accumulation of γH2AX (more DNA damage), resulting in low PARPi resistance in BRCA-expressing cells. Our results reveal KAT2B and histone acetylation as regulators of BRCA2 expression and PARPi responses in BRCA2-expressing CRC cells, providing further insights into molecular prerequisites for targeting BRCA-functional tumors.

## 1. Introduction

Since the development of epidermal growth factor receptor (EGFR) targeted therapy, antiangiogenic drugs, and the use of intense triplet chemotherapy regimens based on fluoropyrimidines, oxaliplatin, and irinotecan, the treatment of colorectal cancer (CRC) has improved significantly [1]. Still, although the overall survival rate of patients with CRC has increased due to these advancements, their impact on CRC treatment has been incremental rather than transformative [2]. Therefore, finding more and targeted strategies to improve disease control in CRC patients and increase their overall survival chances is indispensable.

Subsets of CRC patients with defects in DNA repair pathways have been identified through the molecular and genetic profiling of large CRC datasets, and some of these cases may be accessible for therapeutic targeting. The homologous recombination (HR) repair pathway defect, caused by the germline pathogenic mutations of the breast cancer type 1 susceptibility gene (*BRCA1*), is associated with CRC [3]. This pertains to early-onset CRC [4], but also, in our whole genome analyses of first metastatic lesions compared to corresponding primary CRC lesions and normal background tissues, we found evidence that BRCA-ness molecular patterns increase during CRC progression and metastasis [5,6,7]. Recent studies have revealed that up to 15% of cancer patients have germline or somatic genetic abnormalities in HR repair genes [8,9,10]. These cancers are susceptible to particular DNA-damaging substances, such as platinum compounds and PARP inhibitors (PARPi) [11,12,13].

The European Medicines Agency (EMA) in the European Union and the Food and Drug Administration (FDA) in the United States both authorized olaparib for use as a single agent in December 2014, and olaparib is prescribed to treat breast, ovarian, fallopian tube, peritoneal, pancreas, and prostate cancer [14]. However, factors such as toxicity or an absence of molecularly guided patient selection have impeded the strong development and establishment of PARPi, or their use in combination with other cytotoxic agents, in CRCs [15,16,17,18]. Additionally, for example, a Phase II trial of mono-olaparib therapy containing 33 CRC patients showed that some of these CRC patients with defective DNA repair are still resistant to olaparib [17]. The recovery of the wildtype BRCA is a prominent mechanism of PARPi resistance in BRCA-deficient tumors [19]. Thus, strategies and biomarkers need to be found to decrease the resistance of CRC cells toward olaparib and to improve a molecularly based selection of the most likely responders.

Several studies have discovered histone acetylation to be crucial for genome integrity, replication, and DNA damage response [20,21,22]. Histone acetylation reader proteins can detect signals from histone acetylation on chromosomes. For instance, the bromodomain (BRD), an evolutionary conserved domain that binds the acetylated lysine, allows BRD proteins to identify acetylated histones [23,24]. In combination with PARPi, recent studies have discovered the protein BRD-containing 4 (BRD4) as a synthetic lethal target [25,26]. By inhibiting the transcription of the breast cancer type 1 susceptibility gene (*BRCA1*) and the DNA repair protein homolog 1 (RAD51), BRD4 inhibition has decreased HR in BRCA wildtype tumors [26]. Lysine acetyltransferase 2B (KAT2B), also called the P300/CBP-associated factor (PCAF), belonging to the BRD family, is a lysine acetyltransferase involved in DNA repair, including HR [27], and acetylates core histones to facilitate activation of transcription [28,29,30,31]. Even though more than 50% of human BRD proteins play a role in double-strand break (DSB) repair [30,32,33], little is known about how they affect the transcription of DSB repair molecules.

In the present study, our objective was to define molecular parameters that contribute to olaparib response or resistance in CRC. We report reduced expression of KAT2B after treatment with olaparib. In line with the notion that KAT2B is a transcription-associated protein regulating H3K27 acetylation, we show that histone acetylation affected by KAT2B is a crucial signaling mechanism for the transcription of *BRCA2* in CRCs. Towards this end, KAT2B knockdown or knockout inhibits *BRCA2* transcription by reducing the acetylation of histone H3 at lysine 27 (H3K27ac). Finally, our study shows that inhibiting the critical element of HR repair, KAT2B, decreases PARPi resistance in CRCs through reduced acetylation of H3K27, thus reducing the transcription of *BRCA2*.

## 2. Materials and Methods

The information for all chemicals and antibodies used in this study is summarized in Supplementary Table S1.

### 2.1. Data Download

CRC cell line RNA-seq data were downloaded from the GEO database GSE59857, which included 155 samples. CRC bulk RNA-seq data were from GSE17537, which included 55 samples.

### 2.2. Cell Culture

HCT116, HCT15, SW480, and HEK293T cells were purchased from the American Type Culture Collection (ATCC). HCT116 cells were cultured in McCoy’5A (GIBCO/Thermo Fisher, Berlin, Germany, 16600082) supplemented with 10% fetal calf serum (FCS) (GIBCO/Thermo Fisher, Berlin, Germany, 10500064) and 1% penicillin/streptomycin (PS) (GIBCO/Thermo Fisher, Berlin, Germany, 10378016). HCT15 and SW480 cells were cultured in a Roswell Park Memorial Institute 1640 medium (GIBCO/Thermo Fisher, Berlin, Germany, 21875034) supplemented with 10% FCS (GIBCO/Thermo Fisher, Berlin, Germany, 10500064) and 1% PS (GIBCO/Thermo Fisher, Berlin, Germany, 10378016). HEK293T cells were cultured in a DMEM medium (GIBCO/Thermo Fisher, Berlin, Germany, 11320074) supplemented with 10% FCS and 1% PS (GIBCO/Thermo Fisher, Berlin, Germany, 10378016).

### 2.3. CRISPR/Cas9 Engineering of KAT2B−/− Cells

For the generation of KAT2B−/− HCT116, HCT15, and SW480 cells via CRISPR/Cas9-mediated gene targeting, a combination of two guide RNAs (gRNAs) was used as follows: KAT2B gRNA-1: 5′-CACCGCTTCCTCTGACACATTCTCC-3′ and KAT2B gRNA-2: 5′-CACCGCATATTCATCTGCATATGTG-3′. We inserted the anneal gRNAs into the pSpCas9(BB)−2a-Puro (PX459) V2.0 plasmid as a gift from the Feng Zhang laboratory (Addgene, Watertown, MA, USA, 62988). Recombinant plasmids were transfected into WT HCT116, HCT15, and SW480 cells according to the proportion of 1.5 μgs of plasmids and 7.5 μL of Lipofectamine 2000 (Invitrogen/Thermo Fisher, Berlin, Germany, 11668019). Transfected cells were selected after 24 h of transfection for a further 48 h of applying puromycin (Sigma-Aldrich, Steinheim, Germany, #P8833, 4 µg/mL), and survived cells were transferred to a 96-well plate (one cell per well). Single clones were picked and screened after 20 days via PCR, and Sanger sequencing was performed.

### 2.4. shRNA Transfection

We selected two distinct shRNAs built into the PLKO plasmid obtained from the RNAi consortium (TRC) shRNA library (Broad Institute, United States, TRCN0000207257) to create a stable BRCA2 (shRNA-1: 5′-CCGGGCAGCCATTAAATTGTCCATACTC-GAGTATGGACAATTTAATGGCTGCTTTTTG-3′, shRNA-2: 5′-CCGGGCCTTGA-ATAATCACAGGCAACTCGAGTTGCCTGTGATTATTCAAGGCTTTTTG-3′) or KAT2B (shRNA-1: 5′-CCGGGCAGACTTACAGCGAGTCTTTCTCGAGAAAGA-CTCGCTGTAAGTCTGCTTTTT-3′, shRNA-2: 5′-CCGGGCAGATACCAAACAAG-TTTATCTCGAGATAAACTTGTTTGGTATCTGCTTTTT-3′) knockdown (KD) cell line. The 0.75 × 10^6^ HEK293T cells were seeded in 6-well dishes. After 24 h, 1.5 μg of the control or shRNA plasmid were transfected with the packaging plasmid and the Fu-GENE transfection reagent (Roche, Grenzach-Wyhlen, Germany, 4709705/100). We transferred the virus supernatant onto the HCT116, HCT15, and SW480 cell lines. Twenty-four hours after transfection, the old mix medium was changed to a regular medium. Cells were selected with 10 μg/mL puromycin (Sigma-Aldrich, Germany, #P8833, 10 µg/mL) after seventy-two hours of transfection and then grown in a regular medium with 2 μg/mL puromycin (Sigma-Aldrich, Germany, #P8833, 2 µg/mL).

### 2.5. Immunofluorescence (IF) Staining

For the immunofluorescence staining of cultured cells, 100,000 cells were seeded on coverslips in 24-well plates. Next, cells were washed with PBS (GIBCO/Thermo Fisher, Berlin, Germany, 10010023) and fixed by 4% PFA (Sigma-Aldrich, Steinheim, Germany, 15.812-7) for 10 min. Then, the cells were blocked with a blocking buffer for 1 h and incubated with the primary antibody (Millipore, Darmstadt, Germany, 23103) in a blocking buffer at 4 °C overnight. The next day, cells were incubated with a secondary antibody (Thermo Fisher, Berlin, Germany, Alexa 555) for 2 h at room temperature in the dark, followed by setting with DAPI (Cell Signaling, Danvers, MA, USA, 4083S) for 20 min. Coverslips were washed and mounted in an anti-fade mounting medium. The fluorescence images were acquired using a CONFOCAL microscope (ZEISS, Oberkochen, Germany, LSM 980 with Airyscan 2) with a 25× or 63× objective and analyzed with ImageJ software (National Institutes of Health, Bethesda, MD, USA, RRID:SCR_003070, https://imagej.nih.gov/ij/ (accessed on 1 July, 2021)).

### 2.6. Western Blotting

Cells were cultured in a standard medium (McCoy’5A medium for HCT116, Roswell Park Memorial Institute 1640 medium for HCT15 and SW480). When they reached 70%-80% confluence, the cells were washed and lysed in a radioimmunoprecipitation assay (RIPA) buffer supplemented with protease and phosphatase inhibitors (Thermo Fisher, Berlin, Germany, 78442). The lysate was harvested by scratching. All the experiments were performed on ice. The concentration of protein was measured using the Pierce BCA protein assay kit (Thermo Fisher, Berlin, Germany, 23225). The exact amount of proteins was mixed with a 5X Laemmli sample buffer and boiled at 95 °C for 10 min. The protein was loaded on an SDS-PAGE gel, separated by electrophoresis, and transferred onto the PVDF membrane (BIO-RAD, Neuried, Germany, 1620177). Membranes were stained via Ponceau (Thermo Fisher, Berlin, Germany, A40000279) for 1 min and then blocked at room temperature for 1 h. Then, the membrane was incubated with a primary antibody at 4 °C overnight. Finally, the membranes were incubated with appropriate secondary antibodies (Jackson Immunol., West Grove, PA, USA, 115-035-003, or Jackson Immunol vendor Germany, 115-035-003 or Jackson Immunol, Germany, 111-035-045) at room temperature for 2 h. Immunoreactive bands were observed via chemiluminescence (Vilber, Marne-la-vallée, Ile-de-France, France, FUSION SOLO S, https://www.vilber.com/-fusion-solo-s/ (accessed on 1 March, 2022). Three independent experiments were performed.

### 2.7. RNA Isolation and Q-PCR

RNA was isolated using the TRIzol RNA isolating reagent (Invitrogen/Thermo Fisher, Berlin, Germany, 15596026). cDNA was synthesized using the High-Capacity cDNA Reverse Transcription Kit (Applied Biosystems, Berlin, Germany, 4368814) for quantification of the PCR analysis, and Q-PCR was performed using the SYBR Green PCR master mix (Thermo Fisher, Berlin, Germany, A46012). Cycle numbers were normalized to those of β-actin (QIAGEN GmbH, Düsseldorf, Germany, QT00095431). Q-PCR primers used in this research are shown in the Key resource table.

For RNA-Seq, RNA was isolated using an RNeasy Plus Universal Mini Kit 50 (Qiagen, Düsseldorf, Germany, #73404) according to the manufacturer’s instructions.

### 2.8. RNA-Seq Analysis

RNA was isolated from the HCT116 cell line using the RNeasy Plus Universal Mini kit (Qiagen, Düsseldorf, Germany, #73404) after olaparib (MCE, Monmouth Junction, NJ, USA, AZD2281) or DMSO (Sigma-Aldrich, Steinheim, Germany, 31830) treatment for 24 h. The integrity of the RNA was assessed on a Bioanalyzer 2100 (Agilent, Santa Clara, CA, USA, G2939BA). A library was prepared using the Illumina^®^ Stranded mRNA Prep (Illumina, San Diego, CA, USA, 20040534), and sequencing was performed on a BGISEQ-500 platform (BGI, Yantian, Shenzhen, China, RRID:SCR_017979). RNA-Seq reads were mapped to the hg38 genome reference using STAR (−alignIntronMin 20 −alignIntronMax 500000) (STAR version 2.7.3a, https://github.com/alexdobin/STAR/blob/master/doc (accessed on 1 March, 2022). Read quality was controlled using the FastQC tool (FastQC version 0.12.1, https://github.com/s-andrews/FastQC/releases (accessed on 1 March, 2022)). Bam Tools were used to merge two individual replicates, which can visualize the RNA-seq reads in the Integrative Genomics Viewer (IGV). The bigwig files could be generated using the Bam-Coverage function of deepTools with normalization (-bs 20–smoothLength 40–normalizeUsing RPKM –e 150) (IGV2.8.13, Integra-tive Genomics Viewer, https://igv.org/doc/desktop/ (accessed on 1 March, 2022); BamTools version 2.5.1, USA, https://github.com/pezmaster31/bamtools (accessed on 1 March 2022); deepTools version 3.3.0, Germany, https://deeptools.readthedocs.io/en/develop/ (accessed on 1 March 2022)). DESeq2 was used to quantify and normalize the differential expression (DESeq2 version 1.28.0, USA, http://bioconductor.org/packages/release/bioc/vignettes/DESeq2/inst/doc/DESeq2.html (accessed on 1 March 2022)). Differentially regulated genes were selected using Excel (fold change ≥ 1.5; log2 fold change  ≤−0.58, ≥0.58; *p*-value <  0.05). The gene enrichment pathway analysis was performed using DAVID Bioinformatics Resources 6.8 (DAVID 6.8, USA, https://david.ncifcrf.gov/summary.jsp (accessed on 1 March, 2022)). A volcano plot analysis was performed with the package ggplot2 from R-script according to RPKM normalized values (ggplot2, https://ggplot2.tidyverse.org/ (accessed on 1 March, 2022); R Project for Statistical Computing, RRID:SCR_001-905, https://www.r-project.org/ (accessed on 1 March, 2022)). All data, including publicly available data, were normalized with the same parameters.

### 2.9. Cell Viability Assay

Following the manufacturer’s directions, the CellTiter 96^®^ AQueous One Solution Cell Proliferation Assay (Promega, Walldorf, Germany, G3581) was used to determine the viability of the cells. Three thousand cells per well of the 96-well plate were used for cell seeding. Cells were treated with olaparib or DMSO at different final concentrations of 0 μM, 0.01 μM, 0.1 μM, 1 μM, 10 μM, or 100 μM after one day. Then, the cells were incubated in 20 μL of an MTT solution for 1 h at 37 °C after seven days. At 490 nm, the optical density was measured using the reader (Tecan, Männedorf, Switzerland, Spark^®^ Multimode Microplate Reader). Three replicates were made for each condition.

### 2.10. Clonogenic Survival Assay

In a 6-well plate, 1000 cells were seeded in duplicate. After that, the cells were cultured in a medium with appropriate doses of DMSO or olaparib (final concentration, 2.5 μM for HCT116, 5 μM for HCT15, and 10 μM for SW480). The cell colonies were cultured for one week before being washed three times with PBS, fixed in cold methanol for 15 min, and stained for 15 min at room temperature with Crystal Violet (Sigma-Aldrich, St. Louis, MO, USA, 548-62-9).

### 2.11. Chromatin Immunoprecipitation (ChIP)

ChIP experiments were conducted using a Simple-ChIP Plus Enzymatic Chromatin IP Kit (Magnetic Beads) (Cell Signaling, Germany, #9005). The KAT2B-vector transfected, and KAT2B-KO cells were lysed to obtain DNA–protein complexes, and the samples, after shearing, were immunoprecipitated overnight using an acetyl-H3 antibody (Abcam, Boston, MA, USA, ab4729).

### 2.12. Cell Treatment for Different Assay Conditions

For the DNA damage response assay, olaparib was added to cultured cells to a final concentration of 10 μM/30 μM. For immunoblot assays, the total protein was harvested 8/24/48 h after treatment, and the protein level of γH2Ax (Millipore, Darmstadt, Germany, 23103) was measured using the Western blotting processes as described before. For immunofluorescence staining of γH2Ax (Millipore, Darmstadt, Germany, 23103), cells were washed with PBS and fixed by 4% PFA (Sigma-Aldrich, Steinheim, Germany, 15.812-7) 24 h after treatment. The IF staining was performed following the procedures described in the previous chapter on immunofluorescence staining of cultured cells. At least three independent experiments were performed.

### 2.13. Quantification and Statistical Analysis

Utilizing Prism, statistical computations were performed (Prism, GraphPad, RRID:SCR_002798, https://www.graphpad.com (accessed on 1 July, 2021)). For all datasets obeying normal distribution, statistical significance was determined using the Student’s *t*-test or one-way ANOVA analysis (normality was tested using the Shapiro–Wilk test). If the dataset did not obey a normal distribution, it was tested using an appropriate nonparametric test (Mann–Whitney test or Kolmogorov–Smirnov test). Unless otherwise stated, every experiment was run at least twice. The figures and figure legends show *p*-values and sample sizes. According to the median value, patients were divided into two groups (high KAT2B expression and low KAT2B expression), and survival curves were drawn using the Kaplan–Meier survival analysis. The significance of differences between the groups was determined using the Mantel–Cox log-rank test.

## 3. Results

### 3.1. The Expression of BRCA2 Is Associated with the IC50 for Olaparib in Cultured Colorectal Cancer Cells

**Figure 1:**
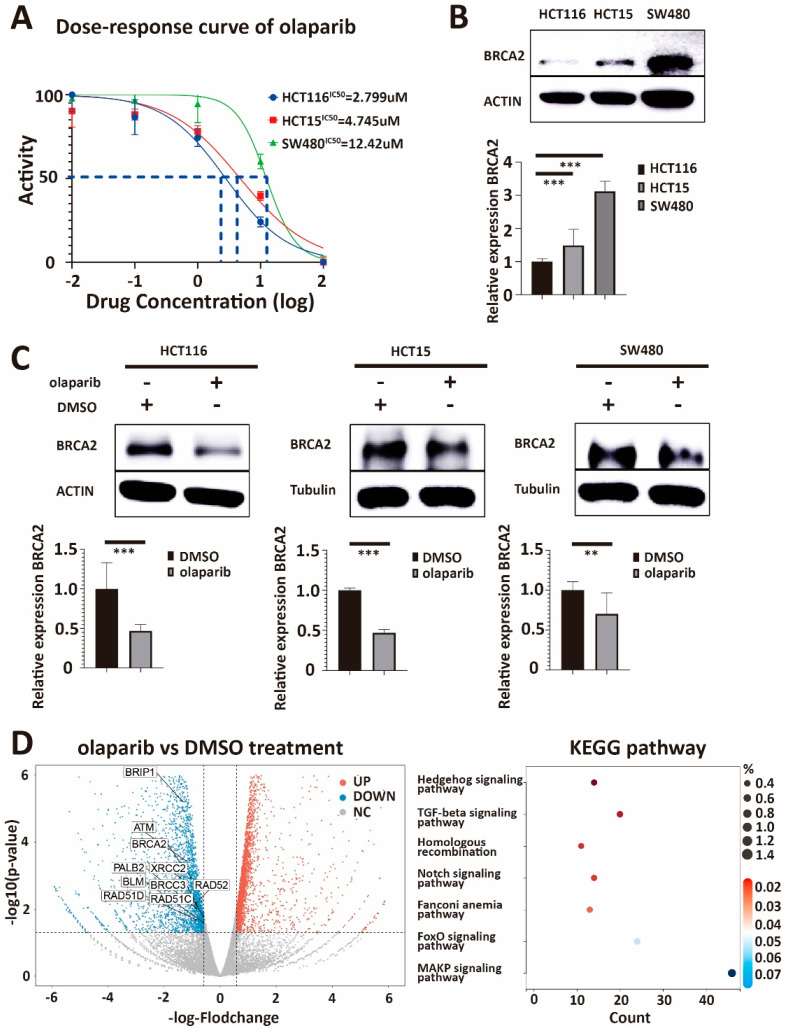
(**A**) IC50 values for olaparib in three colorectal cancer cell lines. The IC50 of HCT116, HCT15, and SW480 are 2.799 μM, 4.745 μM, and 12.42 μM, respectively. IC50 is the half-maximal inhibitory concentration. (**B**) Western blot and q-PCR analysis of lysates derived from HCT116, HCT15, and SW480 CRC cell lines. Western blot and q-PCR show that HCT116 has the lowest expression of BRCA2, the expression level of BRCA2 in HCT15 is intermediate, and SW480 has the relatively highest endogenous expression of BRCA2. (**C**) Western blot and q-PCR analysis of lysates derived from HCT116-DMSO, HCT116-olaparib, HCT15-DMSO, HCT15-olaparib, and SW480-DMSO, SW480-olaparib CRC cell lines. Both Western blot and q-PCR suggest that the expression of BRCA2 in HCT116, HCT15, and SW480 decreases significantly after olaparib treatment. (**D**) Volcano plot showing the log2 fold-change plotted against the log10 *p*-value for DMSO control and olaparib-treated cells. There is a positive correlation between the IC50 of olaparib and the expression of BRCA2 in the three cell lines. DMSO is the negative control, and β-ACTIN and β-tubulin serve as loading controls. β-actin serves as the housekeeping gene (HKG) control in q-PCR. Right graph: KEGG analysis for all the downregulated genes after olaparib treatment 24 Hs. Volcano plot and KEGG analysis also demonstrate that olaparib treatment can reduce the expression of HR-related genes, including BRCA2. Each experiment was repeated at least three times and with two technical replicates, with similar results. Data are presented as the mean ± SD and analyzed using the Student *t*-test. **, *p* < 0.01, ***, *p* < 0.001. The uncropped blots are shown in Figure S8.

To support the role of BRCA2 expression for olaparib sensitivity in colorectal cancer, we first detected the IC50 of olaparib in three different colorectal cancer cell lines: HCT116, HCT15, and SW480 (Figure 1A). Subsequently, we continued to test the BRCA2 expression at mRNA and protein levels with q-PCR and Western blot in these three lines, respectively (Figure 1B). HCT116 had the lowest IC50 for olaparib and BRCA2 expression among the three cell lines. Similarly, SW480 showed the highest IC50 and BRCA2 expression among the three cell lines. The IC50 and BRCA2 expressions were both at a middle level in HCT15. Our results suggest intrinsic olaparib resistance in SW480 compared to HCT116, which appeared to be intrinsically olaparib sensitive. A significant positive correlation between BRCA2 expression and the IC50 of olaparib was found across the three cell lines (*p* = 4.9 × 10^−6^, R = 0.98, Pearson correlation coefficient, Supplementary Figure S1).

To further explore an association between BRCA2 expression and olaparib treatment, we treated HCT116, HCT15, and SW480 with olaparib at a concentration higher than the IC50. We found that BRCA2 expression was decreased in treated groups compared with the respective DMSO group (Figure 1C). In addition, we performed Next Generation RNA Sequencing (NGS) on HCT116 cell lines treated with DMSO or olaparib. After analysis, we found that the expression of *BRCA2, ATM, RAD51C*, and other DNA damage-repair-related genes were significantly downregulated in olaparib-treated groups. In addition, KEGG pathway analysis found that the group of genes downregulated in expression is substantially enriched in genes associated with the homologous recombination pathway (Figure 1D). These results suggest that olaparib treatment decreases *BRCA2* expression and that changes in the expression of further genes involve genes associated with homologous recombination.

### 3.2. BRCA2 Affects the Sensitivity towards Olaparib in Colorectal Cancer Cells

**Figure 2:**
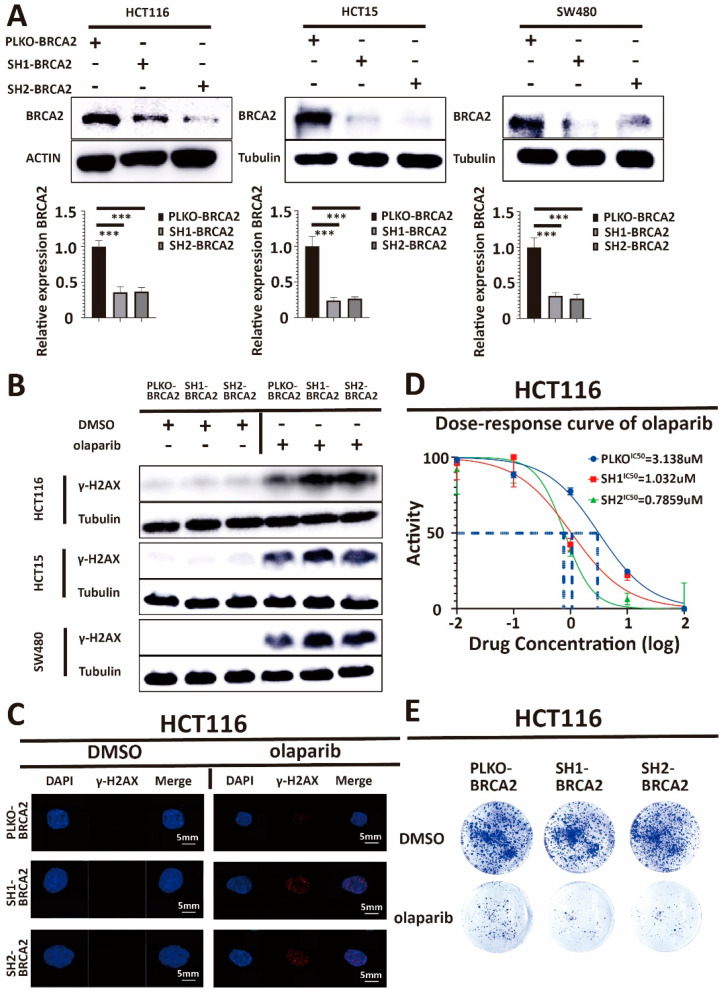
(**A**) Western blot analysis of lysates derived from HCT116-PLKO-BRCA2, HCT116-Sh1-BRCA2, HCT116-Sh2-BRCA2, HCT15-PLKO-BRCA2, HCT15-Sh1-BRCA2, HCT15-Sh2-BRCA2, and SW480-PLKO-BRCA2, SW480-Sh1-BRCA2, SW480-Sh2-BRCA2 CRC cell lines. PLKO is the control. Q-PCR analysis of lysates derived from the same cell lines as in the Western blots above. Western blot and q-PCR show that the expression of BRCA2 in ShRNA-treated cells decreased significantly. β-actin serves as the HKG control in q-PCR. In conclusion, we have successfully obtained BRCA2 knockdown cell lines. (**B**) Western blot analysis of lysates derived from previous cell lines. Western blot and immunofluorescence demonstrate a γ-H2AX accumulation and more DNA damage in BRCA2-knockdown cells after olaparib treatment, compared with control. This indicates that BRCA2 knockdown decreases DNA damage repair after olaparib treatment. PLKO and DMSO are the controls. (**C**) γ-H2AX and DAPI immunofluorescence images in these cell lines. Scale bars, 5 mm. (**D**) Cell viability assay showing that the IC50 of olaparib decreased in HCT116, HCT15, and SW480 after BRCA2 knockdown. (**E**) Clonogenic survival assay demonstrating that clonogenicity is less in HCT116, HCT15, and SW480 cells with BRCA2 knockdown after treatment with the same concentration of olaparib. Taken together, data indicate that BRCA2 knockdown increases the sensitivity of cells toward olaparib treatment. Each experiment was repeated at least three times and with two technical replicates, with similar results. Data are presented as the mean ± SD and are analyzed using the Student *t*-test. ***, *p* < 0.001. The uncropped blots are shown in Figure S9.

Our previous results showed a positive correlation between BRCA2 expression and the IC50 of olaparib. To further support the hypothesis of an association of BRCA2 expression with olaparib sensitivity, we used a BRCA2-ShRNA to construct BRCA2 knockdown cell lines. To demonstrate that the effect of BRCA2 on olaparib treatment was consistent among HCT116, HCT15, and SW480, we used the shRNA for the knockdown of BRCA2 in all three cell lines. The BRCA2-shRNA-plasmid and a lentivirus-plasmid were co-transfected into intermediate host 293T cells, and the resulting supernatant produced by 293T was applied to transfect HCT116, HCT15, and SW480 cells, respectively. Figure 2A shows that BRCA2 expression was lower in shRNA-treated groups than in the PLKO-TRC-vector (PLKO) groups (*p* < 0.001, Student *t*-test). Together, these results suggest that the BRCA2 knockdown cell lines were constructed successfully.

After obtaining stable BRCA2 knockdown cell lines, cultured CRC cells were treated with DMSO and olaparib, respectively. We found that in BRCA2 knockdown cell lines, there was a significant (*p* < 0.001, Student *t*-test) accumulation of γ-H2AX, an established marker of DNA damage, after DNA damage induction. It is known that Serine 139 of H2AX is phosphorylated in a short time after DNA damage, followed by molecular focus formation at DNA break sites, which is evidence of DNA damage [34] (Figure 2B,C, Supplementary Figure S2A,B). Our results are consistent with those previously reported in the literature [11], supporting the hypothesis that BRCA2 knockdown decreases DNA damage repair in cultures of CRC cells after olaparib treatment.

Our Western blot and immunofluorescence results showed more DNA damage after olaparib treatment in BRCA2 knockdown cells (see above). To corroborate these results, we continued to use the cell viability assay and clonogenic survival assay to test the IC50 of olaparib and the efficiency of olaparib in BRCA2-expressing and BRCA2-deficient cell lines. We found that the IC50 of olaparib decreased dramatically after BRCA knockdown, and at the same dose, there were significantly fewer clones in BRCA2-defective cell lines (*p* < 0.001, Student *t*-test) (Figure 2D,E, Supplementary Figure S3A,B). Taken together, this demonstrates that the BRCA2 knockdown increased the sensitivity of CRC cells towards olaparib, strongly supporting the notion that BRCA2 is a crucial influencing factor for the efficiency of olaparib treatment in CRC.

### 3.3. KAT2B Expression Positively Correlates with the IC50 of Olaparib

**Figure 3:**
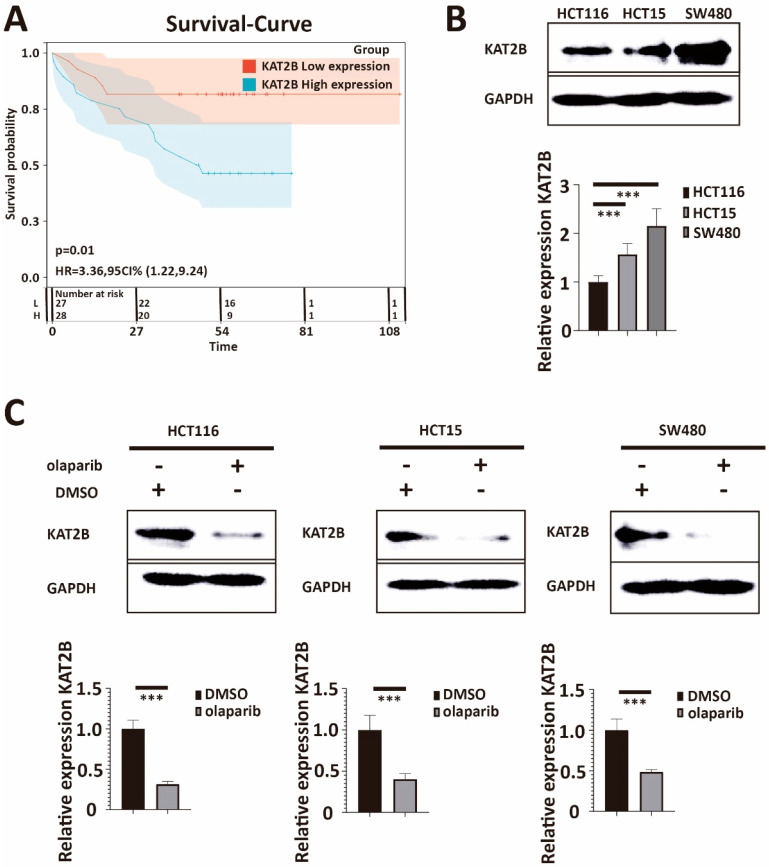
(**A**) Kaplan–Meier curve and Hazard–Ratio from the GSE17537 data set. Kaplan–Meier estimates of survival and Univariate Cox proportional hazards analysis show that higher KAT2B expression is associated with a lower probability of survival. The hazard ratio of KAT2B is higher than 1. Taken together, the data indicate that a high expression of KAT2B is not only a risk factor but also that a high endogenous expression of KAT2B may reduce the survival time of colorectal cancer patients. (**B**) Western blot and q-PCR analysis of lysates derived from HCT116, HCT15, and SW480 cell lines. Western blot and q-PCR illustrate that the expression of KAT2B was lowest in HCT116, followed by HCT15, and highest in SW480. This parallels the IC50 for olaparib among the three cell lines. (**C**) Western blot and q-PCR analysis of lysates derived from HCT116-DMSO, HCT116-olaparib, HCT15-DMSO, HCT15-olaparib, SW480-DMSO, and SW480-olaparib cell lines. Western blot and q-PCR demonstrate that KAT2B expression is significantly decreased in all cells after olaparib treatment. These results imply a negative association between KAT2B expression and olaparib treatment. DMSO is the negative control. GAPDH serves as a loading control. β-actin serves as the HKG in q-PCR. Each experiment was repeated at least three times and with two technical replicates, with similar results. Data are presented as the mean ± SD and analyzed using the Student *t*-test. ***, *p* < 0.001. The uncropped blots are shown in Figure S10.

Our previous RNA-sequencing experiment found that KAT2B is downregulated after olaparib treatment compared to the DMSO group (Figure 1D). Recent studies suggested that KAT2B participates in DNA double-strand damage repair [27,28,29,31], stimulating our interest in studying KAT2B. First, we obtained KAT2B-related survival curves in colorectal cancer patients through Kaplan–Meier analysis from the GSE17537 data set [35]. The Kaplan–Meier survival analysis for KAT2B expression is shown in Figure 3A. Comparison usng the Mantel–Cox log-rank test was significant (*p* = 0.01, log-rank). The univariate Cox proportional hazards analysis for KAT2B expression is shown in Figure 3A (HR = 3.36, 95 CI% [1.22, 9.24]). These results suggest that a high expression of KAT2B is a high-risk factor and may confer a poor survival time in colorectal cancer patients. Encouraged by this, we measured KAT2B expression in our HCT116, HCT15, and SW480 cell lines and found that SW480 (showing the highest IC50 for olaparib) had the highest expression of KAT2B, the expression in HCT15 was in the middle, and HCT116 (showing the lowest IC 50 for olaparib) had the lowest KAT2B expression (Figure 3B), which was consistent with previous results from the GSE59857 series (Supplementary Figure S4A) [36]. Since KAT2B expression parallels the IC50 of olaparib among the three cell lines, these data also showed a positive correlation between the IC50 of olaparib and the expression of KAT2B (*p* = 1.4 × 10^−5^, R = 0.97, Pearson correlation coefficient, Supplementary Figure S4B). In addition, we also found that KAT2B expression was reduced after olaparib treatment according to our Western blot and q-PCR data (Figure 3C). Taken together, these data indicate a similar negative correlation between KAT2B expression and the sensitivity of CRC cells towards olaparib treatment as for BRCA2 expression.

### 3.4. KAT2B Affects the Sensitivity towards Olaparib in Colorectal Cancer Cells

**Figure 4:**
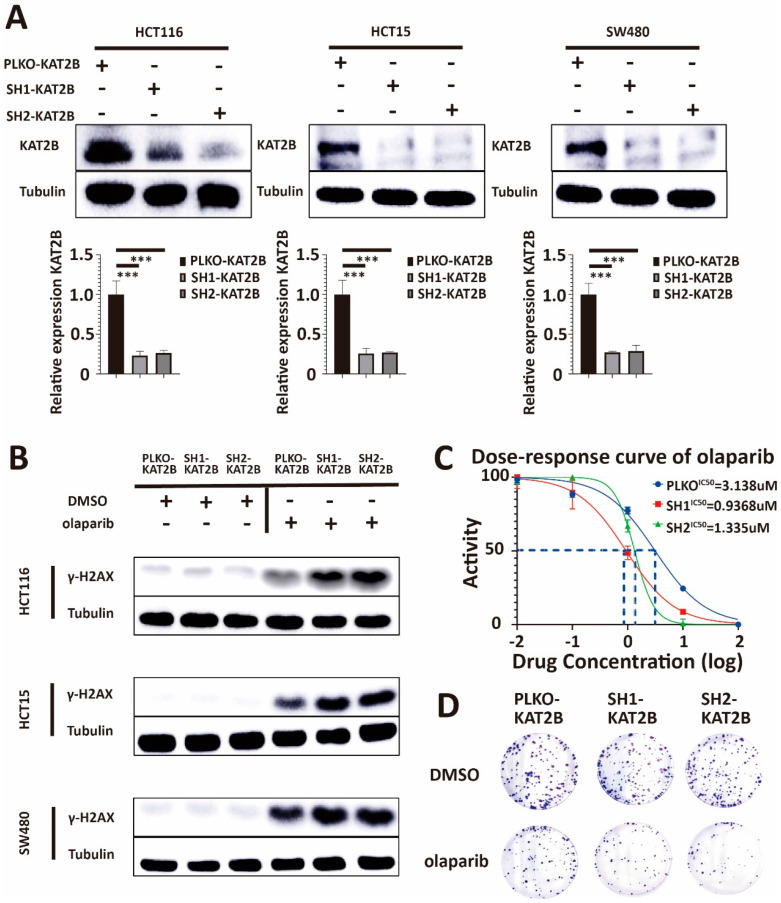
(**A**) Western blot analysis of lysates derived from HCT116-PLKO-KAT2B, HCT116-Sh1-KAT2B, HCT116-Sh2-KAT2B, HCT15-PLKO-KAT2B, HCT15-Sh1-KAT2B, HCT15-Sh2-KAT2B, and SW480-PLKO-KAT2B, SW480-Sh1-KAT2B, and SW480-Sh2-KAT2B CRC cell lines. The upper band in the KAT2B Western blot corresponds to the published main band closely below 100 kDa, the lower band most likely representing non-specific binding [37]. PLKO is the negative control. β-tubulin serves as a loading control. Q-PCR analysis of lysates derived from the same cell lines as given for the Western blot experiments above. Western blot and q-PCR show that, after shRNA treatment, the expression of KAT2B is decreased in HCT116, HCT15, and SW480 cells. These results indicate that we have successfully constructed KAT2B knockdown stable strains. β-actin serves as the HKG in q-PCR. (**B**) Western blot analysis of lysates derived from the previous cell lines. Western blot experiments showed γ-H2AX accumulation and more DNA damage in KAT2B-knockdown cells after olaparib treatment, compared with control. This indicates that KAT2B knockdown decreases DNA damage repair. (**C**) Cell viability assay showing that the IC50 of olaparib decreases in HCT116, HCT15, and SW480 cells after KAT2B knockdown. (**D**) Clonogenic survival assay demonstrating that clonogenicity was less in HCT116, HCT15, and SW480 cells with KAT2B knockdown after treatment with the same concentration of olaparib. This indicates that KAT2B knockdown increases the sensitivity of cells toward olaparib treatment. Each experiment was repeated at least three times and with two technical replicates, with similar results. Data are presented as the mean ± SD and analyzed using a Student *t*-test. ***, *p* < 0.001. The uncropped blots are shown in Figure S11.

To further investigate an association between KAT2B expression and olaparib sensitivity, we knocked down KAT2B by an shRNA against KAT2B. We used an shRNA-plasmid (see Section 2) and a lentivirus-plasmid to transfect 293T cells and the supernatant subsequently produced by 293T cells to transfect HCT116, HCT15, and SW480, respectively. Figure 4A shows that KAT2B expression was lower in the shRNA-treated group compared with the PLKO group (*p* < 0.001, Kolmogorov–Smirnov test). These results suggest that the KAT2B knockdown cell line was constructed successfully.

After we successfully knocked down KAT2B in HCT116, HCT15, and SW480 CRC cells, the cells were treated with DMSO and olaparib to put all the molecules investigated into perspective. First, we found an accumulation of γ-H2AX in KAT2B knockdown cells compared with the control after olaparib treatment (Figure 4B), similar to what we observed in BRCA2 knockdown cells, showing that low KAT2B expression decreases DNA damage repair after olaparib treatment.

Our Western blot results showed that KAT2B knockdown promoted DNA damage, as judged by an accumulation of γ-H2AX (Figure 4B). Therefore, we repeated cell viability and clonogenic survival assays in KAT2B-expressing and KAT2B-deficient cell lines. In these experiments, we found that the IC50 of olaparib decreased significantly with KAT2B knockdown, and the number of clones was severely reduced under the same concentration of olaparib in KAT2B knockdown cells (*p* < 0.001, Student *t*-test) (Figure 4C,D, Supplementary Figure S5A,B). These data demonstrate that KAT2B knockdown renders CRC cells more vulnerable to olaparib treatment, similar to a BRCA2 knockdown.

**Figure 5:**
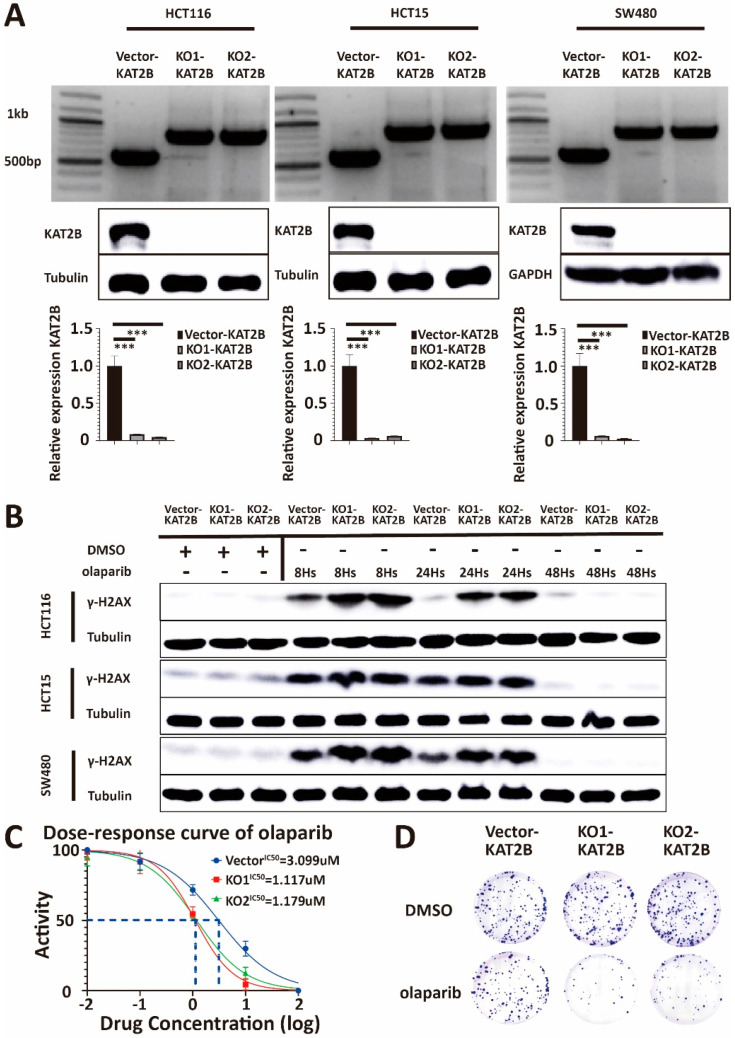
(**A**) Genotyping, Western blot, and q-PCR analysis of lysates derived from HCT116-Vector-KAT2B, HCT116-KO1-KAT2B, HCT116-KO2-KAT2B, HCT15-Vector-KAT2B, HCT15--KO1-KAT2B, HCT15-KO2-KAT2B, and SW480-Vector-KAT2B, SW480-KO1-KAT2B, SW480-KO2-KAT2B cell lines. The results of genotyping show that the bands of the knockout have completely replaced the bands of the wild type. Western blot and q-PCR results show that KAT2B did not express at the protein and mRNA levels. β-actin serves as the HKG control in q-PCR. Therefore, we successfully obtained *KAT2B* knockout cell lines. (**B**) Western blot analysis of lysates derived from the previous cell lines with DMSO and olaparib treatment at different time points. Western blotting shows that the accumulation of γ-H2AX is significantly higher in HCT116, HCT15, and SW480 cells with *KAT2B* knockout than in controls at 8 and 24 h after olaparib treatment, respectively. Collectively, this indicates that the efficiency of DNA damage repair decreased significantly after *KAT2B* knockout. (**C**) Cell viability assay illustrating that the IC50 of olaparib decreased in HCT116, HCT15, and SW480 cells with *KAT2B* knockout. (**D**) Clonogenic survival assay demonstrating that clonogenicity was more inhibited in HCT116, HCT15, and SW480 cells with *KAT2B* knockout at the same concentration of olaparib. In conclusion, *KAT2B* knockout increases the efficiency of olaparib treatment in inhibiting cell proliferation. Vector and DMSO are the controls. β-tubulin and GAPDH serve as a loading control. Each experiment was repeated at least three times and with two technical replicates, with similar results. Data are presented as the mean ± SD and analyzed using the Student *t*-test. ***, *p* < 0.001. The uncropped blots are shown in Figure S12.

Our previous results showed that KAT2B can regulate the sensitivity of colorectal cancer cells toward olaparib. Therefore, we used the CRISPR-Cas9-gene editing system to construct a *KAT2B* knockout cell line. For the preliminary screening of knockout cell lines, we used genotyping for primary genotype identification and further verified loss of mRNA and protein expression with q-PCR and Western blot. The results show that the knockout cell line was established successfully (Figure 5A).

To determine whether a change in KAT2B expression leads to a change in the expression of γ-H2AX, we treated KAT2B knockout and vector control cells with DMSO and olaparib, respectively. We found that the accumulation of γ-H2AX varied at different time points after knocking out KAT2B. As shown in Figure 5B, there was a high level of γ-H2AX expression at both 8 h and 24 h after olaparib treatment in KAT2B knockout cells, implicating that the DNA damage repair, as reflected by changes in γ-H2AX expression, in KAT2B knockout cells may be compromised.

According to the differential expression results of γ-H2AX we obtained before, we again measured the IC50 of olaparib, together with a clonogenic survival assay, in the different cell lines. We found that KAT2B knockout cells were more susceptible to olaparib treatment than the vector cells, which was comparable to the results in BRCA2 knockdown or KAT2B knockdown cells (Figure 5C,D, Supplementary Figure S6A,B).

### 3.5. KAT2B Affects Olaparib Sensitivity through the Regulation of H3K27ac/BRCA2

**Figure 6:**
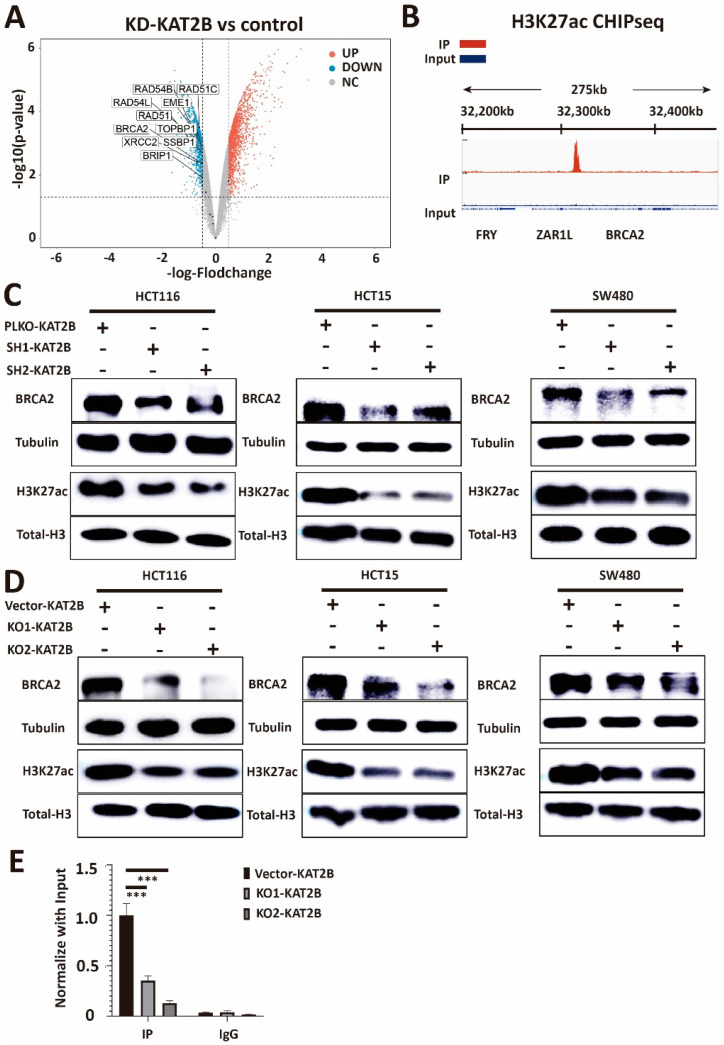
(**A**) Volcano plot showing log2 fold-changes plotted against the log10 *p*-value for control and KAT2B knockdown cells (data from GSE93694). Compared with the control, the volcano plot shows the downregulation of genes related to DNA damage repair in the KAT2B knockdown group. The analysis suggests that KAT2B knockdown reduces many DNA damage repair-related genes, including BRCA2. (**B**) Integrative Genomics Viewer (IGV) for IP and Input in the HCT116 line. IGV shows that the enrichment peak of H3K27ac in the IP group is significantly bound to the promoter of BRCA2 as compared with the Input group, which indicates that H3K27ac may bind to the promoter of *BRCA2* to affect the transcription of BRCA2. (**C**,**D**) Western blot analysis of lysates derived from previous cell lines. Western blotting shows that both KAT2B knockdown and knockout decrease the acetylation of the lysine residue at the N-terminal position 27 of the histone H3 protein (H3K27ac) in HCT116, HCT15, and SW480 CRC cell lines. Moreover, both KAT2B knockdown and knockout also decrease BRCA2 expression in these three cell lines. This suggests that KAT2B regulates H3K27 acetylation and BRCA2 expression. (**E**) ChIP-qPCR analysis of lysates derived from HCT116-Vector-KAT2B, HCT116-KO1-KAT2B, and HCT116-KO2-KAT2B. ChIP-qPCR shows that *KAT2B* knockout significantly reduces the binding of H3K27ac within the promoter of *BRCA2*. Combined with our previous results, we found that both KAT2B knockdown and knockout decrease the expression of BRCA2 by reducing H3K27 acetylation. Vector is the negative control. β-tubulin and Total-Histone3 serve as a loading control. Each experiment was repeated at least three times and with two technical replicates, with similar results. Data are presented as the mean ± SD and analyzed using the Student *t*-test. ***, *p* < 0.001. The uncropped blots are shown in Figure S13.

As we know, KAT2B is a histone acetylation transferase [38], and our previous data showed that KAT2B affects DNA damage repair. To further extend previously described data (GSE93694) [39], GEO2R differential analysis showed that BRCA2, RAD51, and other DNA damage-related molecules were downregulated in KAT2B knockdown cells (Figure 6A). Moreover, analysis of the dataset GSE163820 showed that H3K27ac was significantly enriched in the promoter of *BRCA2* (Figure 6B) [40]. Therefore, we hypothesize that KAT2B may regulate DNA damage repair through the acetylation of histones. Subsequently, we observed that H3K27ac decreased in expression after reducing KAT2B expression (Figure 6C,D), which is consistent with what others reported [41,42,43]. We also observed that the expression of BRCA2 was decreased in both KAT2B knockdown and *KAT2B* knockout cell lines (Figure 6C,D). Subsequently, ChIP-qPCR data showed that the enrichment of H3K27ac in the promoter of BRCA2 was decreased considerably in our *KAT2B* knockout cell lines compared with the control (Figure 6E). This may indicate that KAT2B affects the transcription of *BRCA2* by affecting H3K27ac and that BRCA2 is a downstream target of KAT2B. Also, reduced BRCA2 levels in KAT2B-deficient cells are significantly associated with sensitivity towards olaparib treatment (HCT116, *p* = 5.1 × 10^−6^, R = 0.86; HCT15, *p* = 6.9 × 10^−4^, R = 0.72; SW480 *p* = 1.8 × 10^−3^, R = 0.68, Pearson correlation coefficient, Supplementary Figure S7).

## 4. Discussion

Our present study shows that olaparib resistance is associated with endogenous BRCA 2 expression, and olaparib-resistant CRC cell lines have high endogenous BRCA2. BRCA2 knockdown attenuated the resistance of CRC towards olaparib. In addition, as new cofactors in this scenario, we introduced KAT2B and H3K27ac. Specifically, the knockdown and knockout of KAT2B increases the susceptibility of CRC cells to olaparib (Figure 4 and Figure 5). Mechanistically, we showed that KAT2B changes the transcription of *BRCA2* by modulating acetylated H3K27.

Although olaparib is widely used in the chemotherapy of cancers with BRCA1/2 mutations, drug resistance still poses a significant challenge [44]. Olaparib is a DNA-damaging repair-inhibiting drug, leading to PARP trapping and further generation of double-strand breaks (DSBs), and it has been shown to support the accumulation of DNA damage and to inhibit cell growth by altering regular HR repair [45]. Several studies have revealed that the accumulation of DNA damage might be attributed to the acquired defects or suppression of HR repair molecules [26,46,47]. Alterations of HR repair molecules—such as the phosphoinositide 3-kinase (PI3K) signaling pathway inhibitor BKM120, which can decrease BRCA2 expression—are essential in regulating olaparib sensitivity [46]. Consistently, in this study, the knockdown of BRCA2 reduced the resistance of CRC cells to olaparib (Figure 2D,E) and attenuated the effects on promoting BRCA2-induced HR repair of CRC cells (Figure 2B,C).

Additionally, molecules of the bromodomain and extra-terminal domain (BET) family regulate the transcription of HR repair molecules. Thus, BET inhibitors inhibit the bromodomain (BRD)-containing protein to decrease the transcription of *BRCA1* and *RAD51* [26]. KAT2B, as a member of the BRD family, is a histone acetyltransferase and is associated with homologous recombination repair [27,28,29,30,31]. Previous reports from other laboratories have demonstrated that the acetylation induced by KAT2B significantly modulates the function of H3K27 [41,42,43]. Our present study now adds to this knowledge and shows that KAT2B knockdown and knockout affect the acetylation of H3K27 and the expression of BRCA2 (Figure 6C,D). Mechanistically, our data demonstrate that KAT2B knockdown and knockout attenuate the acetylation of H3K27 and decrease the binding of H3K27ac to the promoter of BRCA2 (Figure 6E). Therefore, the data suggest that less KAT2B-mediated acetylation of H3K27 contributes to a decreased transcription of BRCA2 in olaparib-susceptible CRC cell lines.

Certainly, this study also has its limitations and focuses on in vitro evidence, which still has to be supported by further studies of CRC patient tissues and in vivo models. Nevertheless, as a novel interesting aspect, the study showed that decreased acetylation of H3K27, caused by KAT2B knockdown and knockout, brings about a low expression of BRCA2 in CRC cell lines, which would encourage the development of KAT2B as an additional therapeutic target in this scenario. However, just a few effective small-molecule inhibitors for KAT2B have been produced and analyzed until now. The first study of a selective KAT2B inhibitor involved the synthesis of peptides conjugated with acetyl-CoA, such as H3-CoA-20 [48]. Garcinol is a polyprenylated benzophenone analog that potently inhibits the KAT2B histone acetyltransferase (HAT) domain but is not selective [49]. Isothiazolone derivatives have been suggested as inhibitors of KAT2B with cell-permeability; however, significant off-target activity is still challenging in vivo because of the high chemical reactivity with thiol groups [50]. Therefore, further developmental activities towards a selective and low-off-target KAT2B inhibitor are warranted based on our data. Considering that a promising mechanism of reduced resistance to olaparib may be due to decreased transcription of BRCA2 through the reduced acetylation of H3K27, brought about by low KAT2B expression, we can speculate that low endogenous KAT2B expression might be a representative biomarker for less resistance, possibly including inhibition-specific functional domains such as BRD. Along these lines, designing a KAT2B inhibitor that selectively targets the BRD domain of KAT2B, which can be used in combination with olaparib, will be interesting for future developments. Additionally, since our results demonstrated the impact of KAT2B on the resistance of CRCs to olaparib, KAT2B should be considered for further studies at larger patient sample collectives to be investigated as a biomarker for mono- or combinatorial olaparib treatment. Furthermore, based on our present data, KAT2B could be studied as a biomarker with even higher potential to predict the therapeutic effect of olaparib treatment in CRC patients regardless of whether the patients harbor intrinsic *BRCA*-mutations or not and independent of mutational or molecular patterns suggesting BRCA-ness. If KAT2B could be used as an independent biomarker for olaparib treatment, it would ideally complement our previous findings on the persistence of BRCA-ness mutational signatures in metastatic colorectal cancers [5,6] to better predict the putative response to olaparib in metastatic colorectal cancer patients. Towards this end, this novel *BRCA*-mutation-independent biomarker would be combined with the biomarker BRCA-ness (BRCA-ness referring to the damaged homologous recombination function due to the defects in HR-involved non-*BRCA1/2* genes), to be able to cover prediction for both *BRCA*-mutated and *BRCA*-non-mutated cases. It remains to be seen whether similar options, based on similar molecular observations, could be suggested for other cancer types, especially for breast or ovarian cancer.

In summary, the results of our study reveal that KAT2B-mediated H3K27 acetylation is an essential regulatory mechanism that contributes to the sensitivity of CRC to olaparib in vitro. The study also provides compelling evidence to support the feasibility of targeting KAT2B as an alternative approach for improving the treatment efficacy for olaparib-resistant CRCs.

## 5. Conclusions

**Figure 7:**
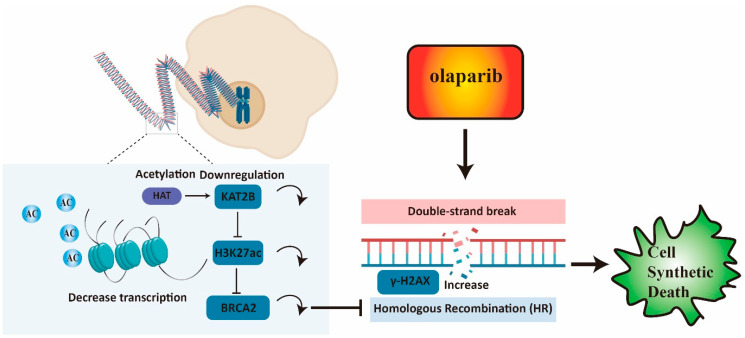
Low expression of BRCA2 reduces homologous recombination repair and increases the accumulation of γ-H2AX. KAT2B knockdown and KAT2B knockout decrease BRCA2 expression by reducing H3K27 acetylation and a reduction of *BRCA2* transcription. A low expression of KAT2B combined with olaparib could be synergistic to inhibit proliferation or even survival in cells by reducing BRCA2 expression.

Our study confirmed that BRCA2 affects olaparib sensitivity in CRC through modulating homologous recombination repair. Moreover, both KAT2B knockdown and KAT2B knockout increase the accumulation of γ-H2AX and DNA damage, decreasing CRC cell resistance toward olaparib treatment. Furthermore, we found that KAT2B may regulate the transcription of *BRCA2* by affecting H3K27ac, suggesting KAT2B inhibition is highly promising to be studied for potential therapeutic synergisms with olaparib. A graphical overview of the results of our paper is given in Figure 7.

## Data Availability

Our RNA-seq results have been deposited at GEO, accession number GEO: GSE246781.This study does not report original codes. Any additional information required to reanalyze the data reported in this paper is available from the lead contact upon request.

## Supplementary Materials

The following supporting information can be downloaded at https://www.mdpi.com/article/10.3390/cancers15235580/s1, Figure S1: Positive correlation between BRCA2 expression and the IC50 for Olaparib in HCT116, HCT15 and SW480 colorectal cancer cells (P = 4.9 × 10^−6^, R = 0.98); Figure S2: (A) γ-H2AX normalized fluorescence intensity in HCT116 cells. (B) Immunofluorescence staining of γ-H2AX and DAPI in defect areas and normalized fluorescence intensity in HCT15 and SW480 cells; Figure S3: (A) IC50 for Olaparib in HCT15 and SW480 cells after knockdown of BRCA2. (B) Colony formation assay of HCT116, HCT15, and SW480 cells after knockdown of BRCA2, and normalized clones in these different cell lines. Each experiment was repeated at least three times with similar results; Figure S4. (A) Relative transcript per million (TPM) of KAT2B in HCT116, HCT15, and SW480 CRC cells from the GSE59857 series. (B) Positive correlation between KAT2B expression and the IC50 for Olaparib in HCT116, HCT15, and SW480 colorectal cancer cells (P = 1.4 × 10^−5^, R = 0.97); Figure S5: (A) IC50 of Olaparib in HCT15 and SW480 after knockdown of KAT2B. (B) Colony formation assay of HCT116, HCT15, and SW480 cells after knockdown of KAT2B, and normalized clones in these different cell lines; Figure S6: (A) IC50 of Olaparib in HCT15 and SW480 after knockout of *KAT2B*. (B) Colony formation assay of HCT116, HCT15, and SW480 cells after knockout of *KAT2B*, and normalized clones in these different cell lines; Figure S7: Positive correlation between BRCA2 expression and Olaparib IC50 in HCT116, HCT15 and SW480 colorectal cancer cells with KAT2B knockdown or knockout (HCT116, P = 5.1 × 10^−6^, R = 0.86; HCT15, P = 6.9 × 10^−4^, R = 0.72; SW480 P = 1.8 × 10^−3^, R = 0.68); Figures S8–S13: Original blots of Figures 1–6; Table S1: Overview on reagents used in this work, including sources and reference numbers of the respective companies/catalogues.
