# Supplementary material for: Epigenetically Downregulated Breast Cancer Gene 2 through Acetyltransferase Lysine Acetyltransferase 2B Increases the Sensitivity of Colorectal Cancer to Olaparib
Source: Cancers (Basel). 2023 Nov 25;15(23):5580. doi: 10.3390/cancers15235580 (PMC10705808; doi:10.3390/cancers15235580)
Supplement: Supplementary file 1 [file cancers-15-05580-s001.zip › Supplementary Figures S1-S7 and Table S1.pdf]

Supplementary figures

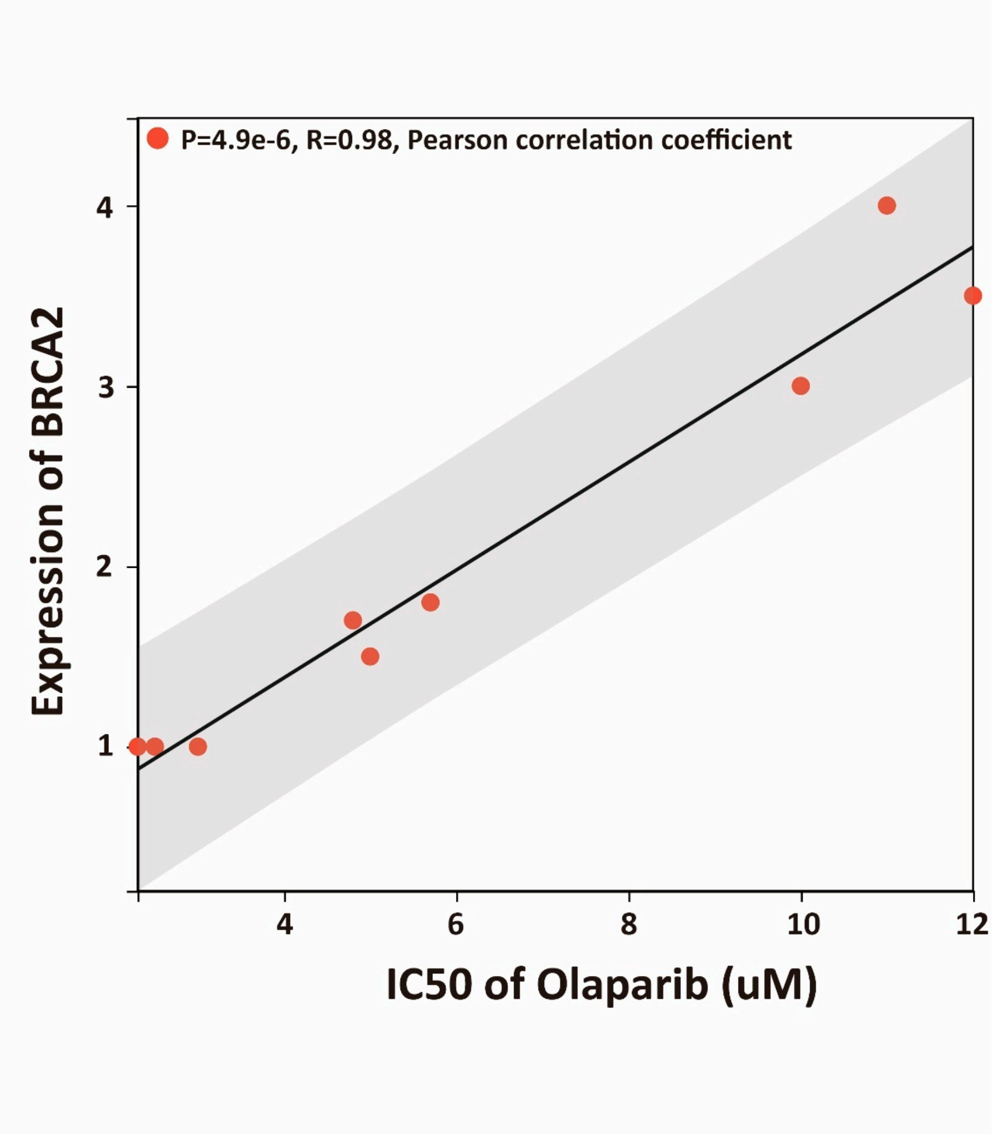

Supplementary figure S1. Positive correlation between BRCA2 expression and the IC50 for Olaparib in HCT116, HCT15 and SW480 colorectal cancer cells (P=4.9e-6, R=0.98).

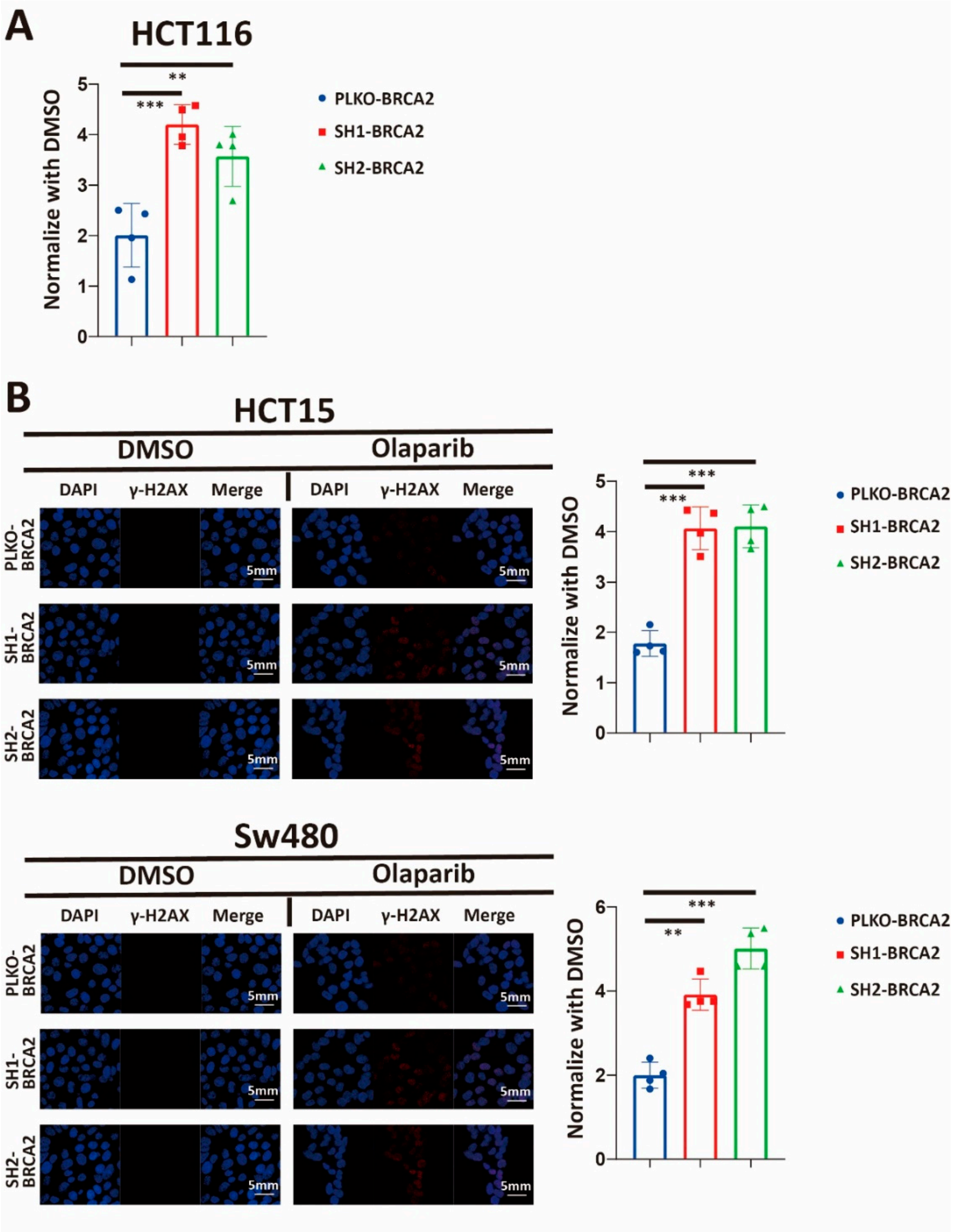

Supplementary figure S2. (A) γ-H2AX normalized fluorescence intensity in HCT116 cells. (B) Immunofluorescence staining of γ-H2AX and DAPI in defect areas and normalized fluorescence intensity in HCT15 and SW480 cells. The scale bar is 5mm. Each experiment was repeated at least three times with similar results. Data are presented as the mean ± SD and analyzed by the Student t-test. **, $P<0.01$; ***, $P<0.001$.

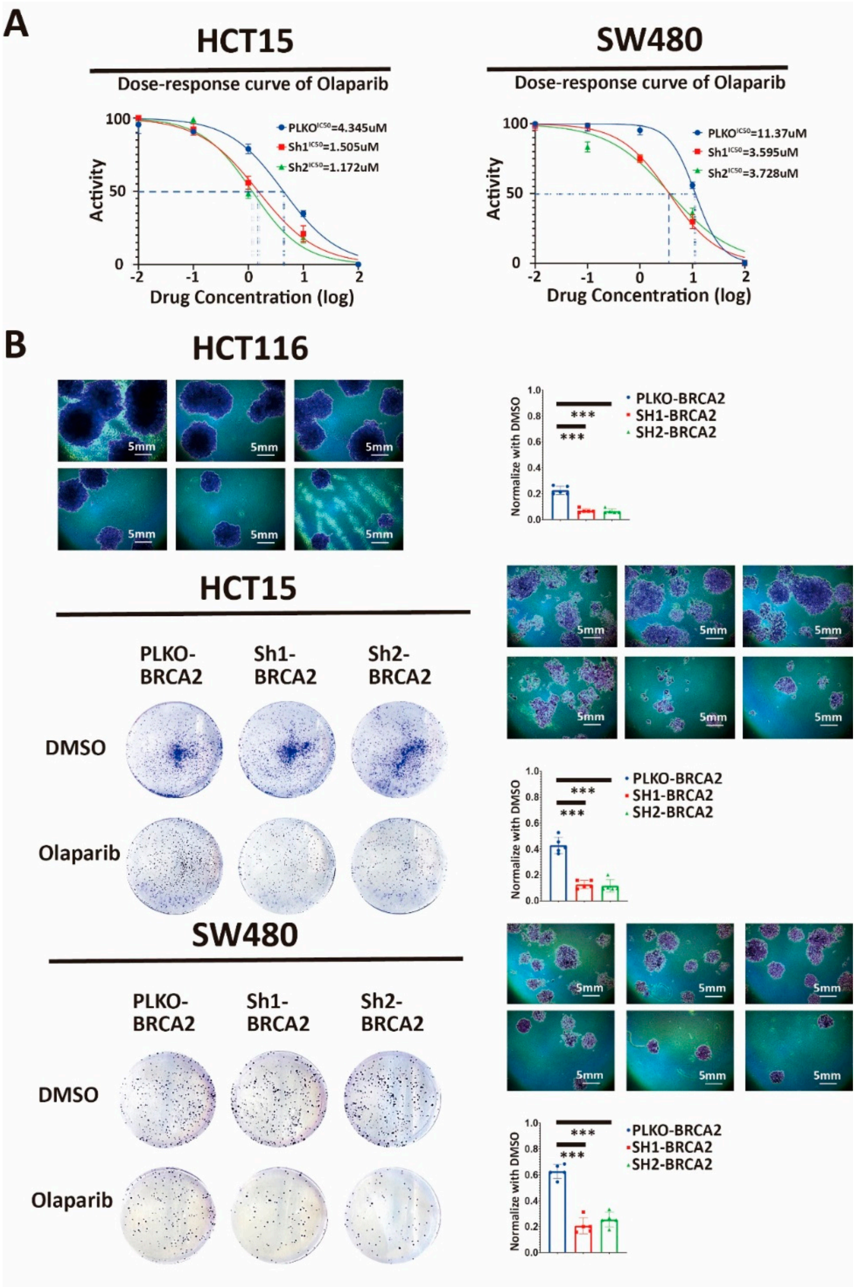

Supplementary figure S3. (A) IC50 for Olaparib in HCT15 and SW480 cells after knockdown of BRCA2. (B) Colony formation assay of HCT116, HCT15, and SW480 cells after knockdown of BRCA2, and normalized clones in these different cell lines. Each experiment was repeated at least three times with similar results. The scale bar is 5mm. Data are presented as the mean ± SD and analyzed by Student t-test. ***, $P<0.001$.

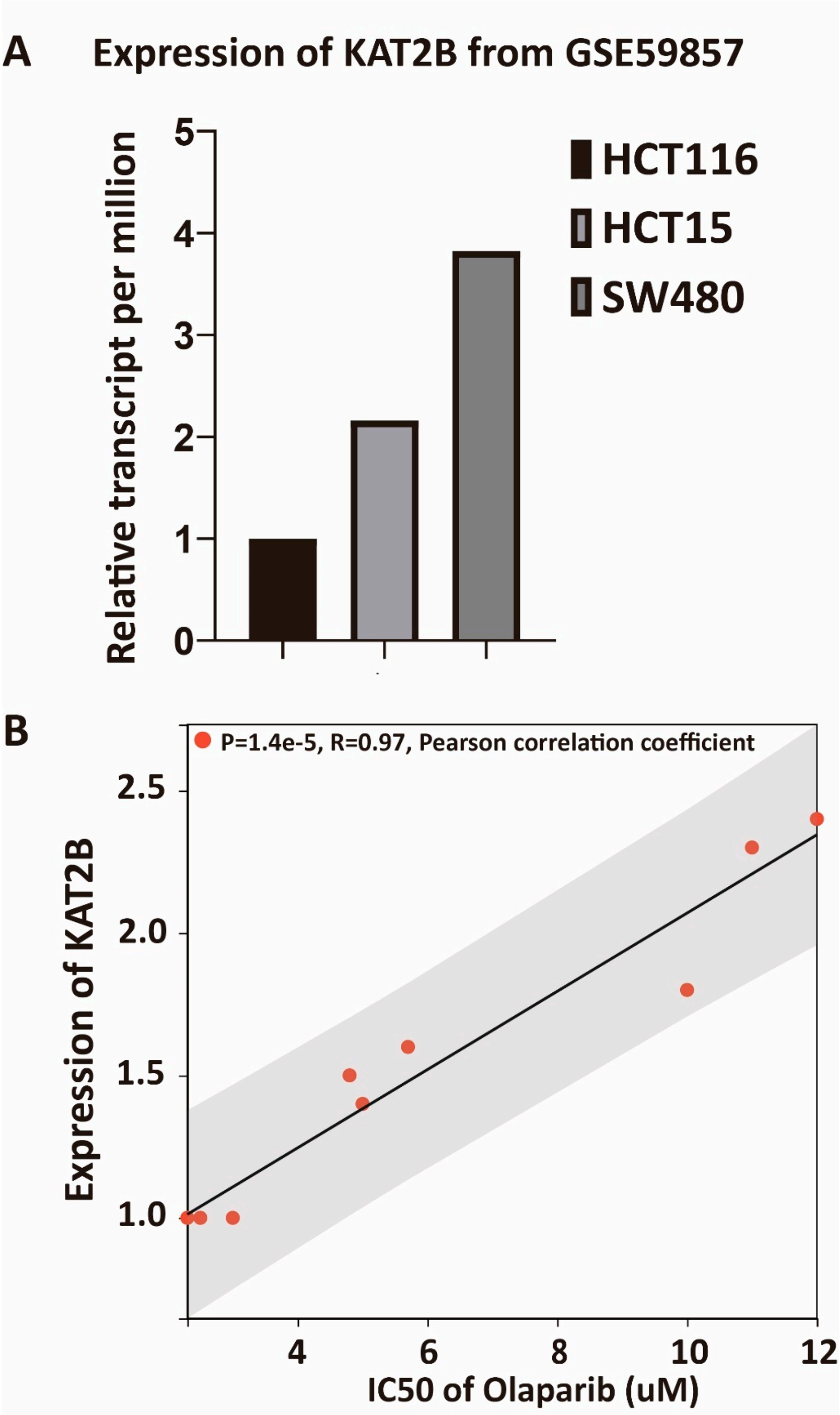

Supplementary figure S4. A, Relative transcript per million (TPM) of KAT2B in HCT116, HCT15, and SW480 CRC cells from the GSE59857 series. B, Positive correlation between KAT2B expression and the IC50 for Olaparib in HCT116, HCT15, and SW480 colorectal cancer cells (P=1.4e-5, R=0.97).

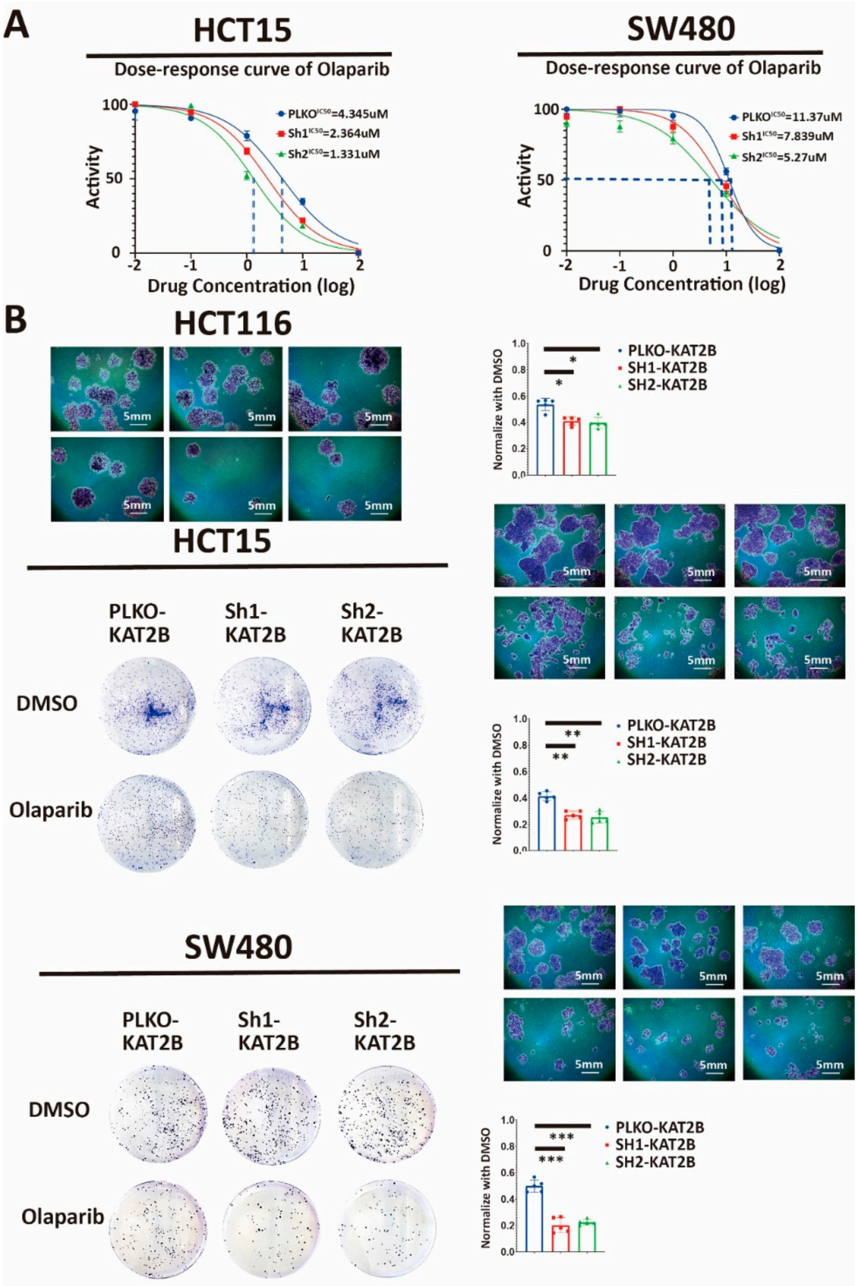

Supplementary figure S5. (A) IC50 of Olaparib in HCT15 and SW480 after knockdown of KAT2B. (B) Colony formation assay of HCT116, HCT15, and SW480 cells after knockdown of KAT2B, and normalized clones in these different cell lines. The scale bar is 5mm. The scale bar is 5mm. Each experiment was repeated at least three times with similar results. Data are presented as the mean ± SD and analyzed by Student t-test. *, P<0.05; **, P<0.01; ***, P<0.001.

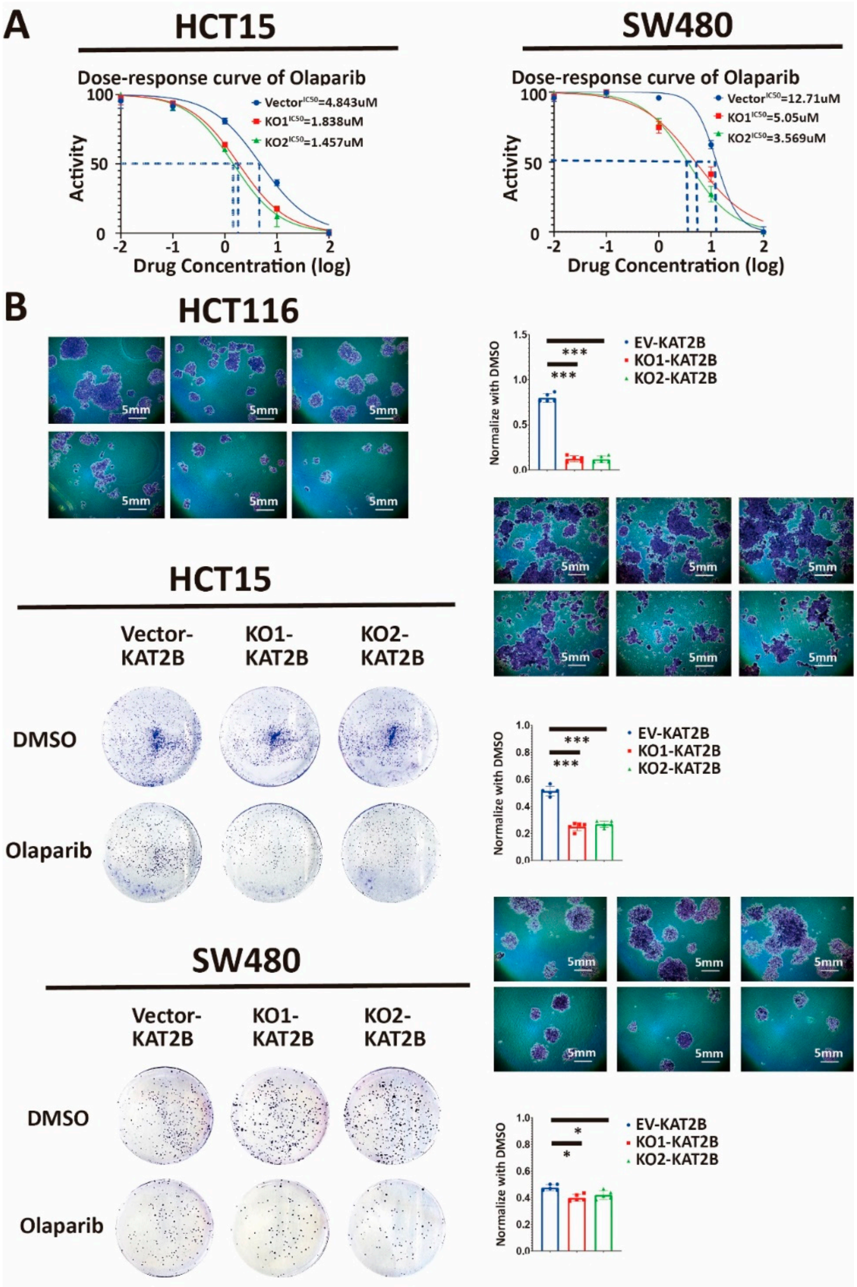

Supplementary figure S6. (A) IC50 of Olaparib in HCT15 and SW480 after knockout of KAT2B. (B) Colony formation assay of HCT116, HCT15, and SW480 cells after knockout of KAT2B, and normalized clones in these different cell lines. The scale bar is 5mm. Each experiment was repeated at least three times with similar results. Data are presented as the mean ± SD and analyzed by Student t-test. *, P<0.05; **, P<0.01; ***, P<0.001.

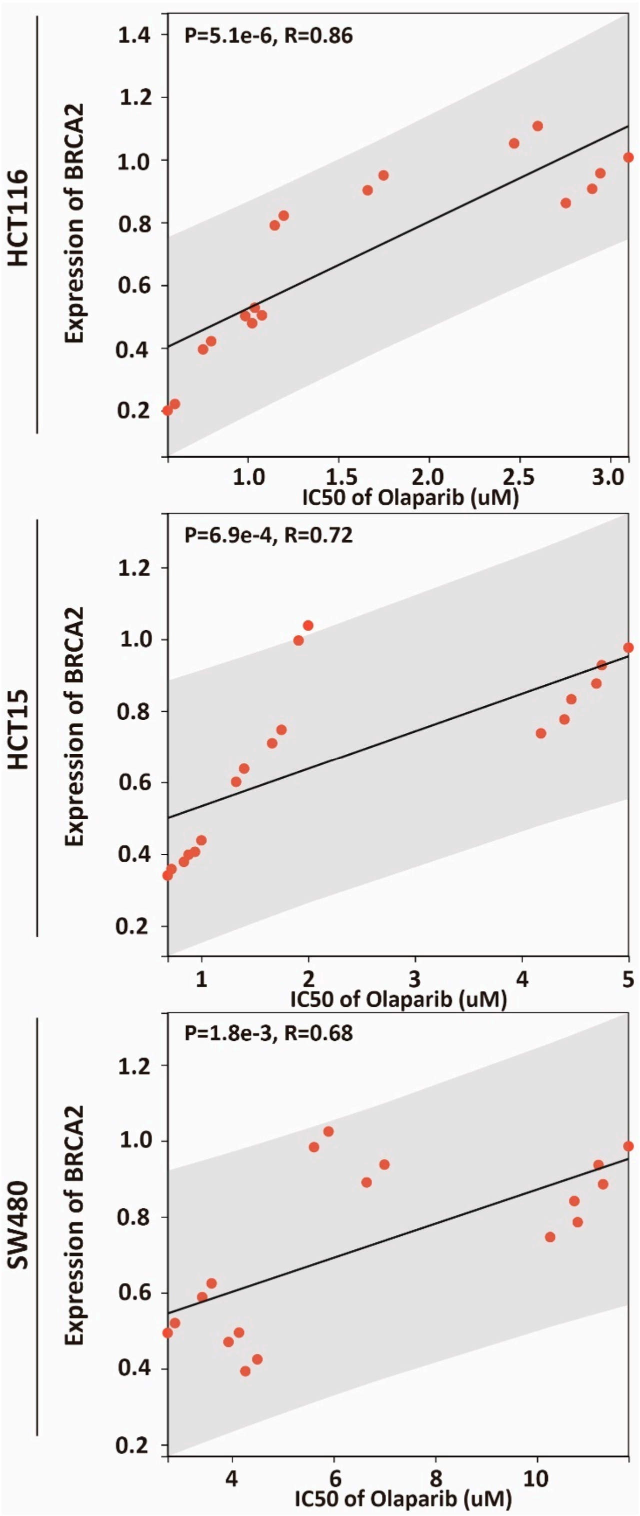

Supplementary figure S7. Positive correlation between BRCA2 expression and Olaparib IC50 in HCT116, HCT15 and SW480 colorectal cancer cells with KAT2B knockdown or knockout (HCT116, P=5.1e-6, R=0.86; HCT15, P=6.9e-4, R=0.72; SW480 P=1.8e-3, R=0.68).

Supplementary table S1

Substance	Company	Location	Reference number
ddH2O	Veolia	Germany	PURELAB® flex 1
Phosphate-buffered saline (PBS)	GIBCO/ Thermo Fisher	Germany	10010023
2-Log DNA ladder (0.1-1.0 kb)	NEB	Germany	#N3200L
Albumin fraction V (BSA)	Roth	Germany	8076.2
Agarose, low melt	Roth	Germany	6351.2
Noble agar	Sigma-Aldrich	Germany	A5431
Ammonium persulfate (APS) ($(NH_4)_2S_2O_8$)	Sigma-Aldrich	Germany	A3678-25G
Ampicillin sodium salt	Sigma-Aldrich	Germany	A99548-25G
Chloroform ($CHCl_3$)	Roth	Germany	Y015.1
Dimethyl sulfoxide (DMSO) ($(CH_3)_2SO$)	Sigma-Aldrich	Germany	31830
dNTP (Nucleoside triphosphate) Set 1	Roth	Germany	178.1
DTT (Dithiothreitol) ($C_4H_{10}O_2S_2$)	Roth	Germany	6908.3
Dry milk, non-fat milk	BIO-RAD	Germany	170-6404
Immun-Blot PVDF Membrane	BIO-RAD	Germany	1620177
EGTA (ethylene glycol tetraacetic acid) ($C_{14}H_{24}N_2O_{10}$)	Roth	Germany	3054.2
Ethanol-denatured (CH_3CH_2OH)	Roth	Germany	K928.3
Ethidium bromide solution ($C_{21}H_{20}BrN_3$)	Sigma-Aldrich	Germany	E1510-10ML
Ethylenediaminetetraacetic acid (EDTA) ($C_{10}H_{16}N_2O_8$)	Sigma-Aldrich	Germany	E5134-250G
Roche X-tremeGENE HP DNA Transfection Reagent	Roche	Germany	6366236001

Roche FuGENE® HD Transfection Reagent	Roche	Germany	4709705/100
Formamide (CH_3NO)	Fluka	Germany	47670
37% formaldehyde solution	Sigma-Aldrich	Germany	F8775-25ML
Glycerol ($C_3H_8O_3$)	Roth	Germany	3783.1
Glycine (NH_2CH_2COOH)	Sigma-Aldrich	Germany	15527
Isopropanol (C_3H_8O)	Roth	Germany	6752.4
LB-agar (Lennox)	Roth	Germany	X965.2
LB-medium (Lennox)	Roth	Germany	X964.2
Methanol (CH_3OH)	Roth	Germany	4627.5
Magnesium chloride ($MgCl_2$)	Sigma-Aldrich	Germany	M2393-500G
Magnesium sulfate ($MgSO_4$)	Fischer	Germany	M120.37
TEMED (Tetramethylethylenediamine) ($(CH_3)_2NCH_2CH_2N(CH_3)_2$)	Roth	Germany	2367.1
Nonidet P-40 (NP-40)	Fluka	Germany	74385
Paraformaldehyde (PFA) ($OH(CH_2O)_nH$ ($n = 8 - 100$))	Sigma-Aldrich	Germany	15.812-7
Phenol red ($C_{19}H_{14}O_5S$)	Sigma-Aldrich	Germany	P0290
Phosphatase inhibitor Cocktail set V	Calbiochem	Germany	524632
PMSF (Phenylmethylsulfonyl fluoride) ($C_7H_7FO_2S$)	Serva	Germany	32395
Prestained protein molecular weight marker	Fermentas	Germany	#SM0441
Protease inhibitor cocktail set I	Calbiochem	Germany	535142
Puromycin	Sigma-Aldrich	Germany	#P8833
Sodium dodecyl sulfate (SDS) ($NaC_{12}H_{25}SO_4$)	Sigma-Aldrich	Germany	L4390-100G
Sodium phosphate monobasic monohydrate	Sigma-Aldrich	Germany	53522-1KG

($NaH_2PO_4 \cdot H_2O$)			
Sodium phosphate dibasic dodecahydrate ($Na_2HPO_4 \cdot 12H_2O$)	Sigma-Aldrich	Germany	04273
TRIzol® reagent	Invitrogen/ Thermo Fisher	Germany	15596026
Triethanolamine ($C_6H_{15}NO_3$)	Sigma-Aldrich	Germany	T1377-500ML
TRIS (tris(hydroxymethyl)aminomethane) ($(HOCH_2)_3CNH_2$)	Roth	Germany	4855.2
Sodium Citrate Dihydrate	Sigma-Aldrich	Germany	W302600
Triton X-100 ($(C_{14}H_{22}O(C_2H_4O)_n)$)	Sigma-Aldrich	Germany	X100-500ML
Tween 20 EP, NF	Sigma-Aldrich	Germany	T2700-500ML
Xylene	Roth	Germany	CN80.2
Hematoxylin Solution	Sigma-Aldrich	Germany	GHS116
Eosin Y Solution	Sigma-Aldrich	Germany	HT110216
Crystal violet Solution	Sigma-Aldrich	Germany	HT901
ProLong ™ Gold Antifade Mountant	Thermo Fisher	Germany	P36930
DMEM 4.5g GlutMax	GIBCO/ Thermo Fisher	Germany	21885025
RPMI 1640	GIBCO/ Thermo Fisher	Germany	21875034
DMEM/F12 (1:1)	GIBCO/ Thermo Fisher	Germany	11320074
Fetal Calf Serum (FCS)	GIBCO/ Thermo Fisher	Germany	10500064
100x Pen/Strep	GIBCO/ Thermo Fisher	Germany	10378016
100x L-glutamine	GIBCO/ Thermo Fisher	Germany	25030081

McCoy’5A	GIBCO/ Thermo Fisher	Germany	16600082
RNAiMax	Invitrogen/ Thermo Fisher	Germany	2418078
Opti-MEM	GIBCO/ Thermo Fisher	Germany	31985-062
β-mercaptoethanol	Sigma-Aldrich	Germany	M3148-100ML
Glycerol	Roth	Germany	3783.1
Bromophenol Blue Sodium Salt	Sigma-Aldrich	Germany	B8026-25G
Lipofectamine 2000	Invitrogen/ Thermo Fisher	Germany	11668019
Olaparib	Medchemexpress (MCE)	Germany	AZD2281
**Kit**	**Supplier**	**Location**	**Reference number**
2x SYBRGreen	Thermo Fisher	Germany	A46109
GenElute™ gel extraction kit	Sigma-Aldrich	Germany	NA1111-1KT
GenElute™ HP plasmid miniprep kit	Sigma-Aldrich	Germany	PLN70-1KT
GenElute™ HP plasmid midiprep kit	Sigma-Aldrich	Germany	PLD35-1KT
High-Capacity cDNA Reverse Transcription Kit	Thermo Fisher	Germany	4368814
RedTaq® ReadyMix TM PCR Reaction Mix	Sigma-Aldrich	Germany	R2523-100RXN
Thermo Scientific™ Pierce™ BCA™ Protein Assay	Thermo Fisher	Germany	23225
RNeasy Plus Universal Mini Kit 50	QIAGEN GmbH	Germany	73404
Dynabeads™ M-280 sheep anti-mouse IgG	Invitrogen/ Thermo Fisher	Germany	11202D
Dynabeads™ M-280 sheep anti-rabbit	Invitrogen/	Germany	11203D

IgG	Thermo Fisher			
Halt™ Protease and Phosphatase Inhibitor Single-Use Cocktail (100X)	Thermo Fisher	Germany	78442	
CellTiter 96® AQueous One Solution Cell Proliferation Assay (MTS)	Promega	Germany	G3581	
**Antibodies**	**Company**	**Location**	**Cat. No.**	**Dilution**
BRCA2	Cell Signaling	Germany	mAb#10741	WB 1:2000
KAT2B	Cell Signaling	Germany	mAb #3378	WB 1:2000
H3K27ac	Abcam	Germany	ab4729	WB 1:2000<br>ChIP 1:10
H3	Abcam	Germany	ab1791	WB 1:2000
β-Actin	Cell Signaling	Germany	93473	WB 1:2000
β-Tubulin	Abcam	Germany	ab18207	WB 1:2000
Glycerinaldehyd-3-phosphat-Dehydrogenase (GAPDH)	Abcam	Germany	ab8245	WB 1:2000
γH2Ax	Millipore	Germany	23103	WB 1:2000<br>IF 1:250
HPR-Anti-Mouse secondary antibody	Jackson Immunol	Germany	115-035-003	WB 1:5000
HRP-Anti-Rabbit secondary antibody	Jackson Immunol	Germany	111-035-045	WB 1:5000
Rabbit Alexa 488	Invitrogen/ Thermo Fisher	Germany	A-21206	IF 1:500
DAPI	Cell Signaling	Germany	4083S	1:10000
**Primers**	**Sequence (5'-3')**	**Company**	**Location**	**Reference number**
q-PCR KAT2B	AGGAAAACCT-	QIAGEN	Germany	QT00092267

forward	GTGGTTGAAGG	GmbH		
q-PCR KAT2B reverse	CAGTCTTCGT-TGAGATGGTGC	QIAGEN GmbH	Germany	QT00092267
q-PCR ACTB forward	CATGTACGTTG-CTATCCAGGC	QIAGEN GmbH	Germany	QT00095431
q-PCR ACTB reverse	CTCCTTAATGT-CACGCACGAT	QIAGEN GmbH	Germany	QT00095431
q-PCR BRCA2 forward	CACCCACCCTT-AGTTCTACTGT	Metabion	Germany	220908B010D06 (lot number)
q-PCR BRCA2 reverse	CCAATGTGGTC-TTTGCAGCTAT	Metabion	Germany	220908B010D06 (lot number)
ChIP-q-PCR BRCA2 forward	CTTTTGTCTCT-GCCAACCCC	Metabion	Germany	220908B010H06 (lot number)
ChIP-q-PCR BRCA2 reverse	GACAGCTGGG-AGGGAAGTTA	Metabion	Germany	220908B010H06 (lot number)
ChIP-q-PCR BRCA2 forward	CACTGAGAAA-TACCCGCAGC	Metabion	Germany	220908B010F06 (lot number)
ChIP-q-PCR BRCA2 reverse	AAATCTGTCC-CCTCACGCTT	Metabion	Germany	220908B010F06 (lot number)
gRNA1- KAT2B forward	CACCGCCACATCC-AATACTATTCCC	Metabion	Germany	221027B038E02 (lot number)
gRNA1- KAT2B reverse	AAACGGGAATAG-TATTGGATGTGGC	Metabion	Germany	221027B038E02 (lot number)
gRNA2- KAT2B forward	CACCGCTTCCTC-TGACACATTCTCC	Metabion	Germany	221027B038C02 (lot number)
gRNA2- KAT2B reverse	AAACGGAGAATG-TGTCAGAGGAAGC	Metabion	Germany	221027B038C02 (lot number)
gRNA3- KAT2B forward	CACCGCATATTC-ATCTGCATATGTG	Metabion	Germany	221027B038A02 (lot number)

gRNA3- KAT2B reverse	AAACCACATATG-CAGATGAATATGC	Metabion	Germany	221027B038A02 (lot number)
gRNA4- KAT2B forward	CACCGCGCAAC-ACTGTTTTGTCACT	Metabion	Germany	221027B038G01 (lot number)
gRNA4- KAT2B reverse	AAACAGTGACA-AAACAGTGTTGCGC	Metabion	Germany	221027B038G01 (lot number)
Genotyping for cells forward-1	ACCCTCTTGA-CATGGTGGAG	Metabion	Germany	221027B038D03 (lot number)
Genotyping for cells forward-2	CTTTGCAGCA-GACAGTGAGG	Metabion	Germany	221027B038C03 (lot number)
Genotyping for cells forward-3	GGATAGTGAG-TGAAAGCGGC	Metabion	Germany	221027B038B04 (lot number)
Genotyping for cells reverse-1	AAGGGAGGA-ACGGAGAAAGG	Metabion	Germany	221027B038B03 (lot number)
Genotyping for cells reverse-2	ACCCACCAAA-TTCCAGGACT	Metabion	Germany	221027B038A03 (lot number)
Genotyping for cells reverse-3	TTTCTGGGAT-CACTGCGACT	Metabion	Germany	221027B038A04 (lot number)
