# Supplementary figures and images for: Epigenetically Downregulated Breast Cancer Gene 2 through Acetyltransferase Lysine Acetyltransferase 2B Increases the Sensitivity of Colorectal Cancer to Olaparib
Source: Cancers (Basel). 2023 Nov 25;15(23):5580. doi: 10.3390/cancers15235580 (PMC10705808; doi:10.3390/cancers15235580)

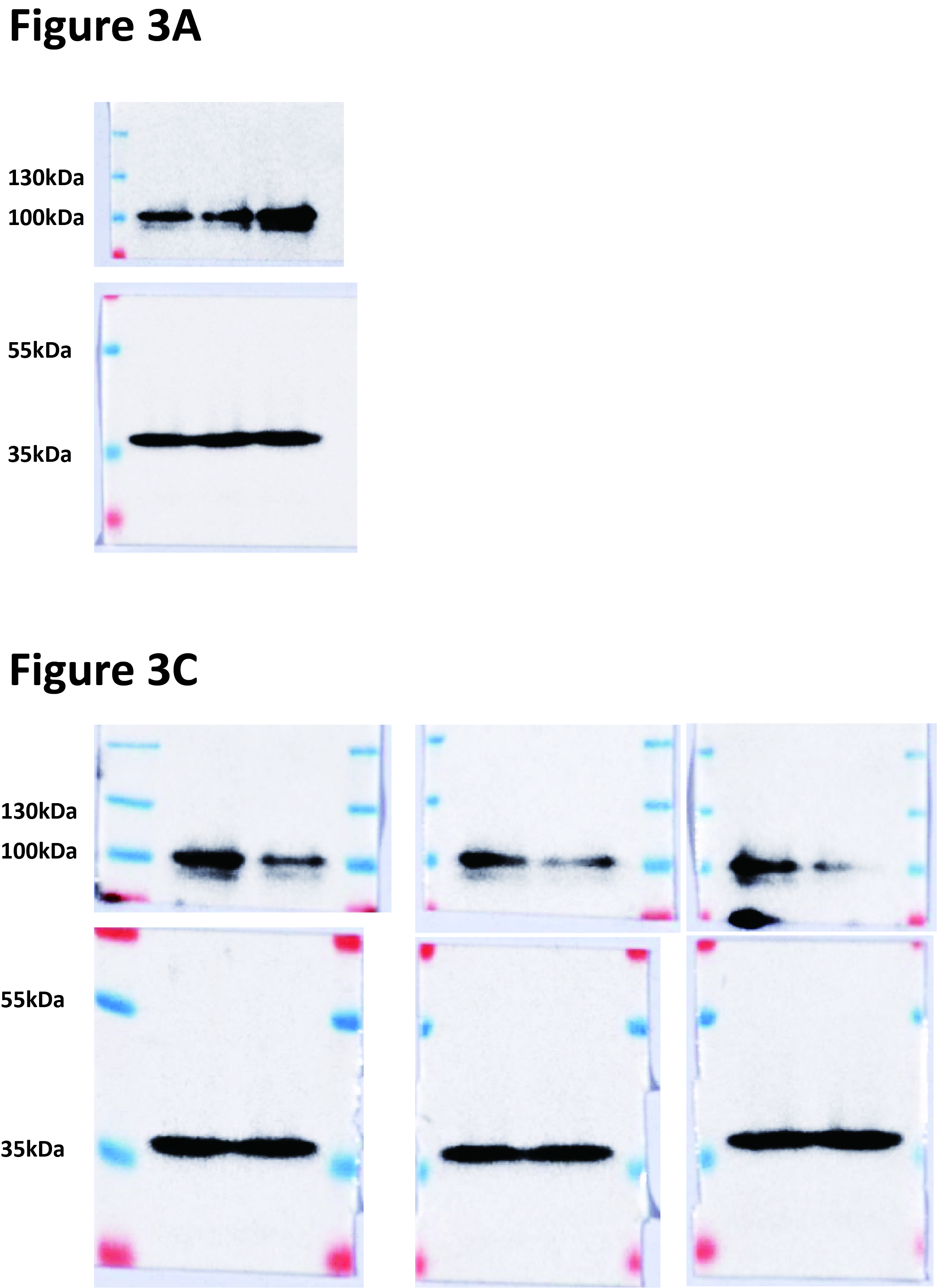

Supplement: Supplementary file 1 [file cancers-15-05580-s001.zip › Figure S10-original blots of Figure 3.jpg]

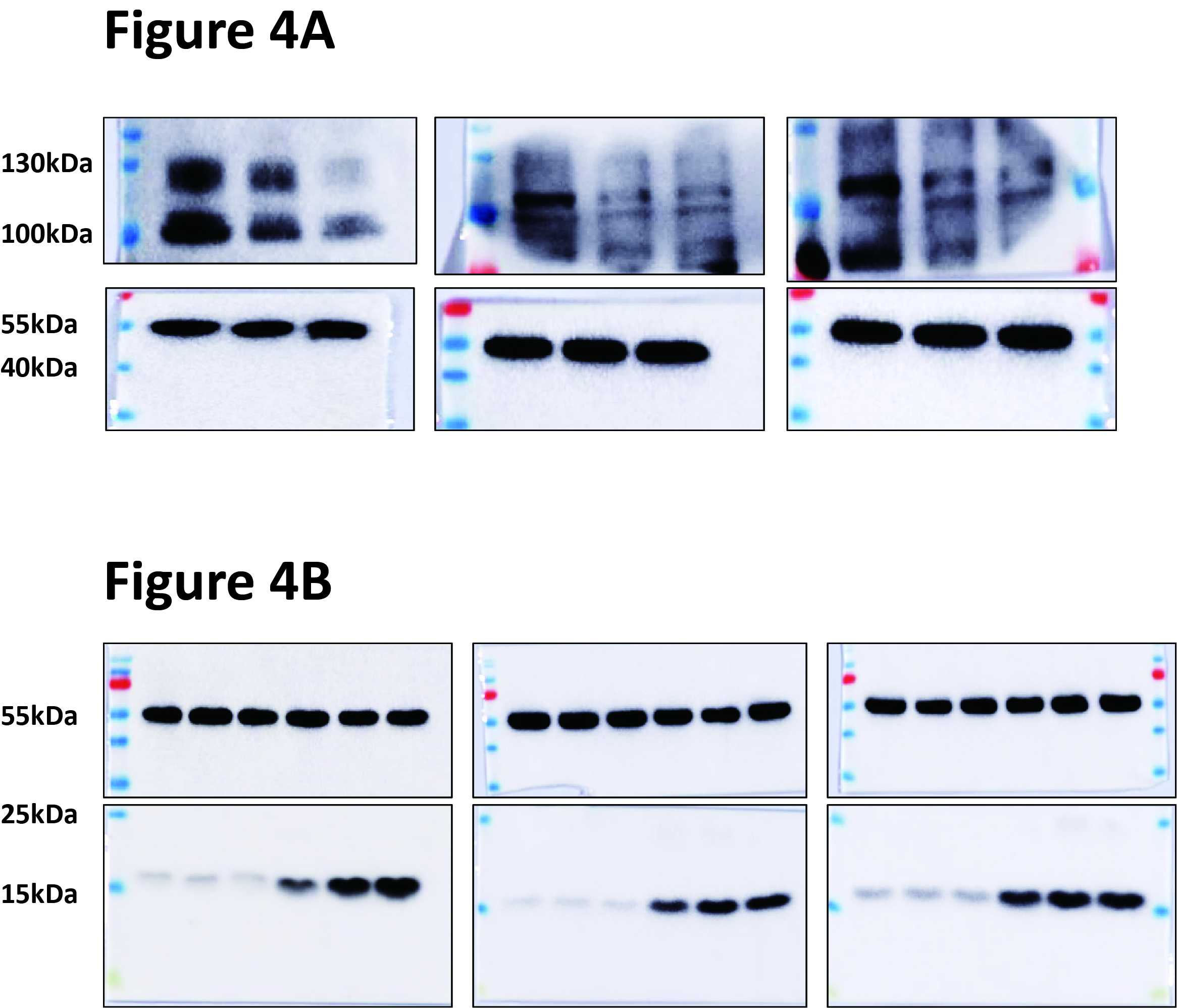

Supplement: Supplementary file 1 [file cancers-15-05580-s001.zip › Figure S11-original blots of Figure 4.jpg]

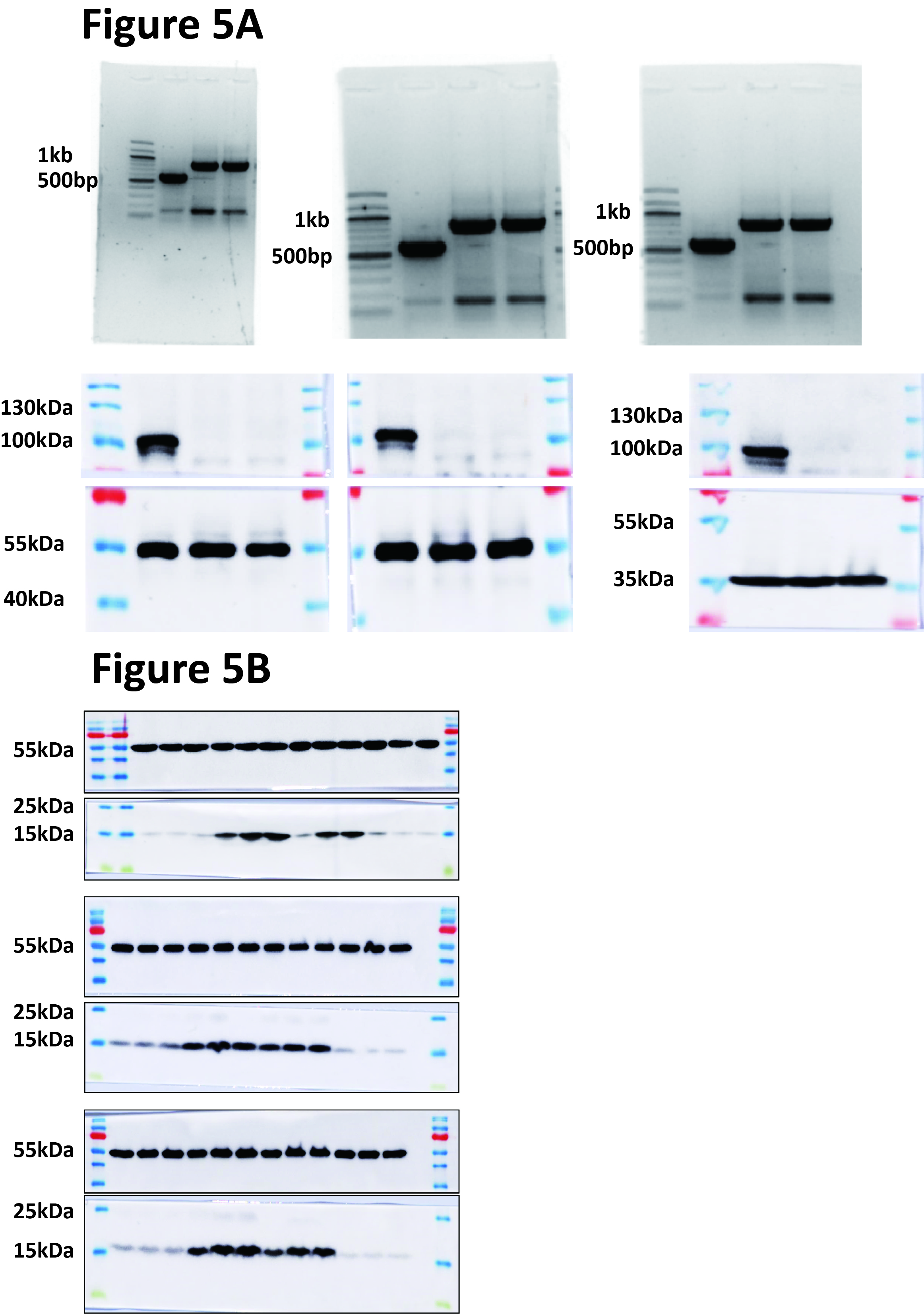

Supplement: Supplementary file 1 [file cancers-15-05580-s001.zip › Figure S12-original blots of Figure 5.jpg]

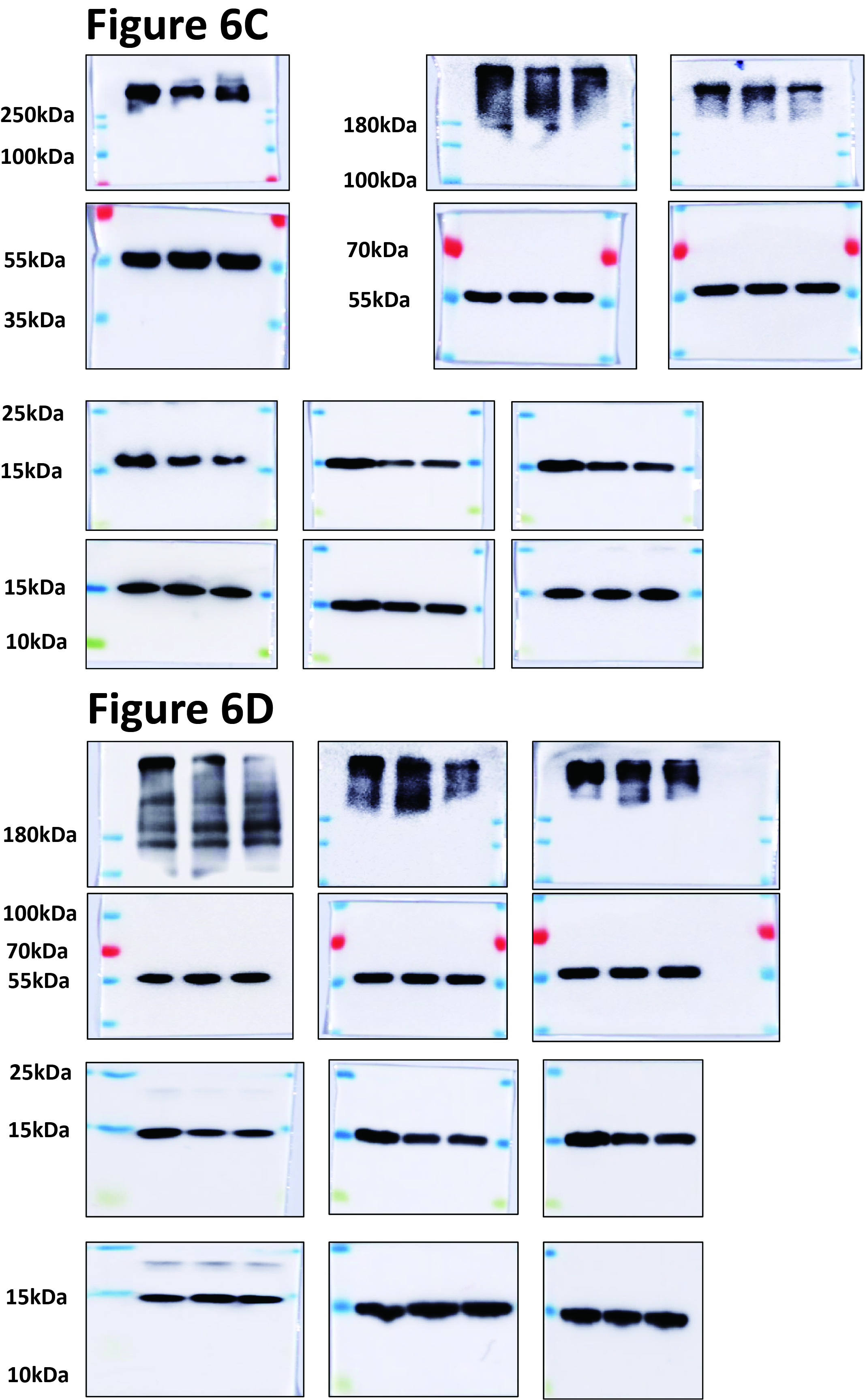

Supplement: Supplementary file 1 [file cancers-15-05580-s001.zip › Figure S13-original blots of Figure 6.jpg]

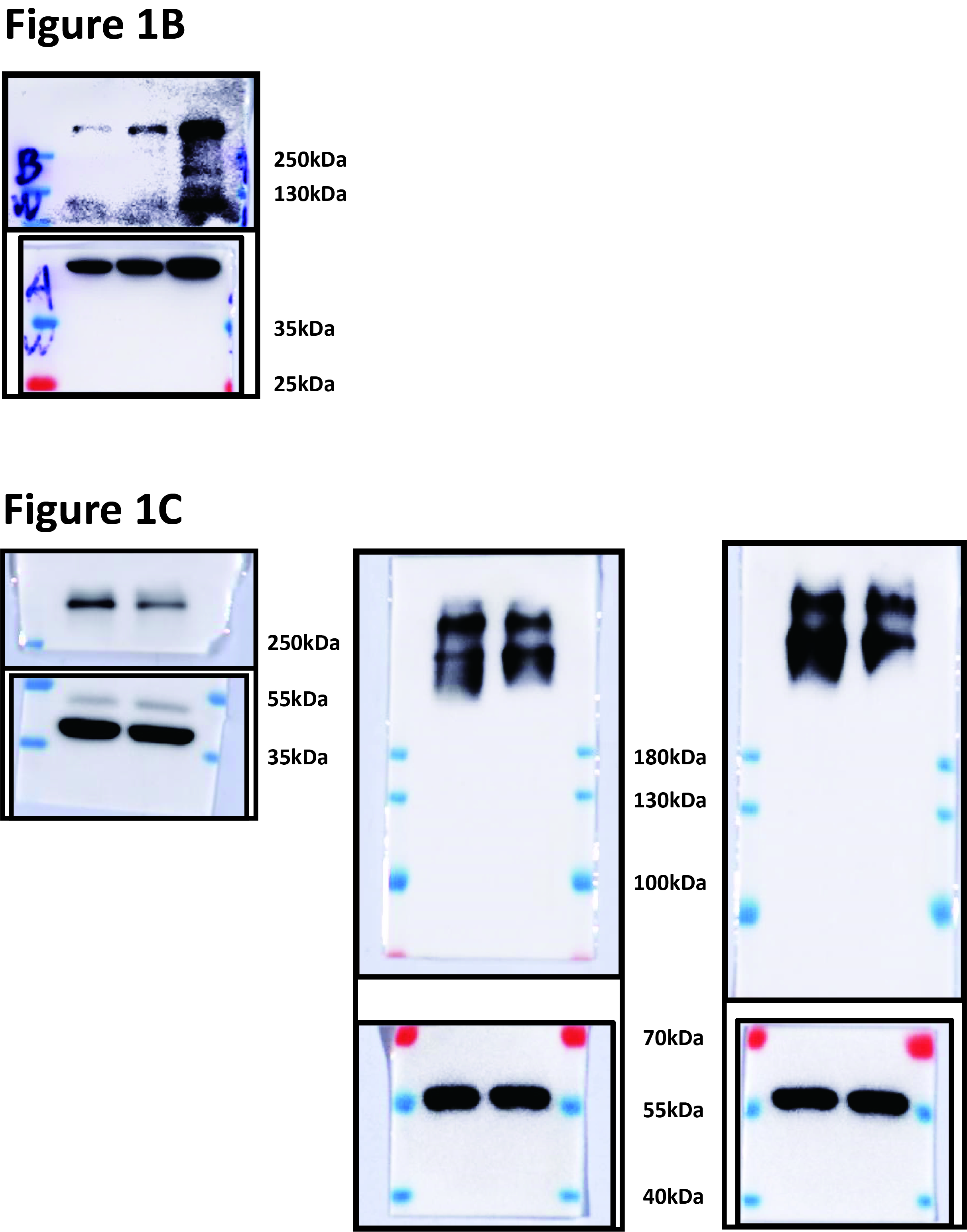

Supplement: Supplementary file 1 [file cancers-15-05580-s001.zip › Figure S8-original blots of Figure 1.jpg]

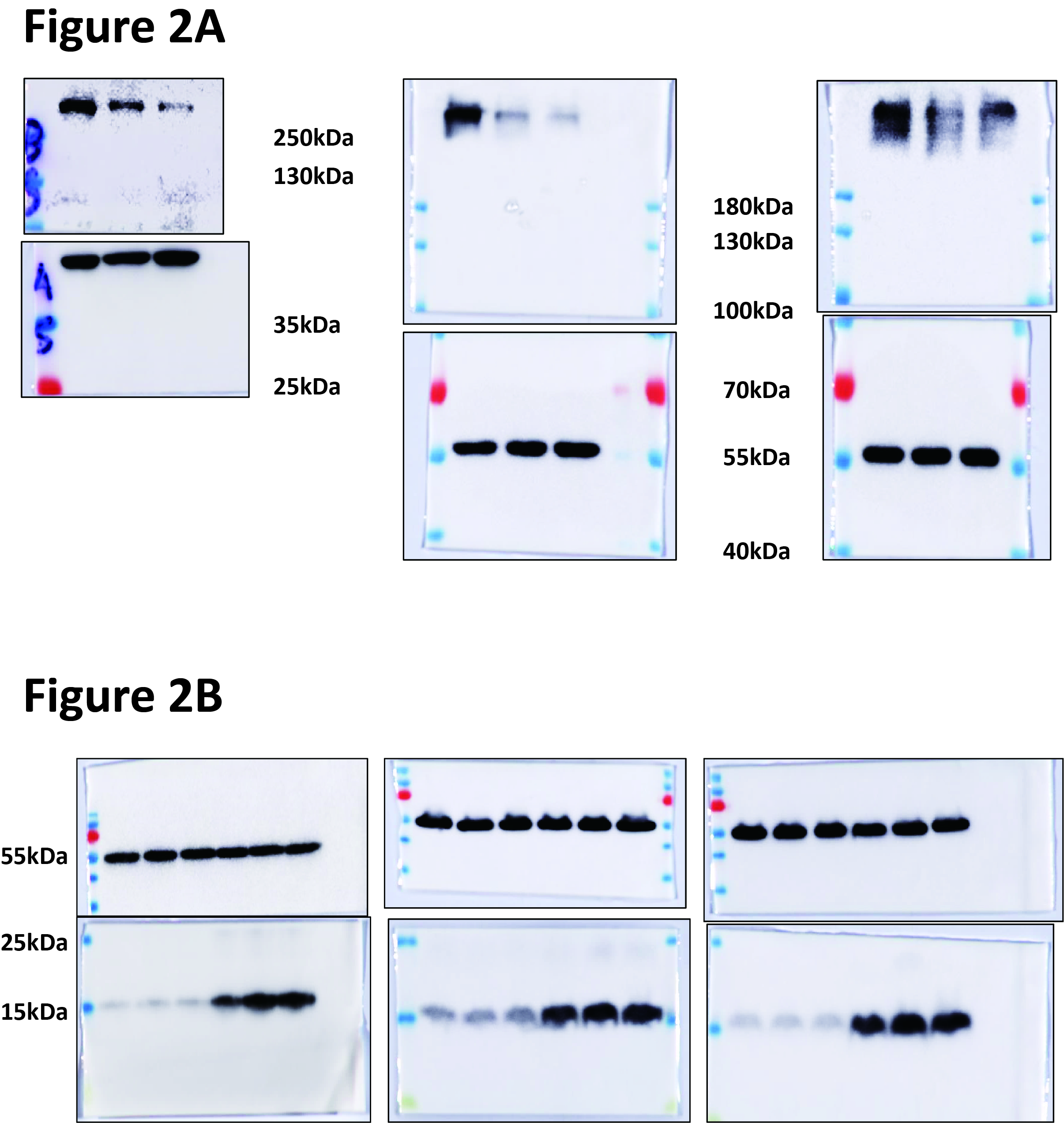

Supplement: Supplementary file 1 [file cancers-15-05580-s001.zip › Figure S9-original blots of Figure 2.jpg]
